# Integrated transcriptomic analysis and machine learning identify AURKA and CENPM as candidate biomarkers in focal segmental glomerulosclerosis

**DOI:** 10.1371/journal.pone.0361466

**Published:** 2026-11-10

**Authors:** Xiaomei Lin, Guofang Huang, Qing Sun, Xikui Zhang

**Affiliations:** 1 Fujian University of Traditional Chinese Medicine, Fuzhou, Fujian Province, China; 2 Rehabilitation Hospital Affiliated to Fujian University of Traditional Chinese Medicine, Fuzhou, Fujian Province, China; 3 The Second Affiliated Hospital of Fujian University of Traditional Chinese Medicine, Fuzhou, Fujian Province, China; Centre de Recherche en Biologie cellulaire de Montpellier, FRANCE

## Abstract

**Background:**

Focal segmental glomerulosclerosis (FSGS) is a leading cause of steroid-resistant nephrotic syndrome and progression to end-stage renal disease, but existing therapeutics and diagnostics are limited. Therefore, reliable diagnostic biomarkers that contribute to FSGS therapy and diagnostics are urgently needed.

**Methods:**

Gene expression data for FSGS (GSE104948; 37 samples: 16 FSGS vs 21 controls) were obtained from GEO. A weighted gene co-expression network analysis (WGCNA) was employed. Key genes within significant modules were identified using Support Vector Machine Recursive Feature Elimination (SVM-RFE), random forest (RF), and cytoHubba Maximal Clique Centrality (MCC). Functional enrichment analyses were executed, while diagnostic accuracy was evaluated by receiver operating characteristic (ROC) analysis. Immune cell infiltration (ICI) levels were quantified using CIBERSORT, and validation was conducted with an independent external dataset (GSE129973; 20 FSGS vs 20 controls).

**Results:**

The green-yellow module demonstrated robust association with the FSGS phenotype, from which 39 candidate genes were selected. Integration of RF, SVM-RFE and cytoHubba analyses pinpointed two primary hub genes: AURKA, which was recovered consistently across cross-validation folds, and CENPM, a less consistently recovered candidate. Functional enrichment underscored the involvement of these genes in pivotal biological processes, particularly mitosis and regulation of the cell cycle. Analysis of immune infiltration revealed significant shifts in the immune microenvironment (IME) of FSGS, correlating elevated expression of AURKA and CENPM with altered ICI patterns. ROC curve analyses showed the diagnostic performance of these genes, with AURKA achieving an AUC of 0.95 and CENPM 0.94 in the training dataset (independent validation: AURKA AUC 0.797, CENPM AUC 0.720).

These results showed a consistent direction of change between FSGS patients and healthy controls in an independent validation cohort.

**Conclusion:**

Using a combined analytical approach involving WGCNA, machine learning (ML) algorithms, and protein-protein interaction (PPI) networks, this study identified AURKA and CENPM as exploratory tissue-based candidate biomarkers for FSGS, with AURKA being the more robustly supported of the two; both require functional validation and independent confirmation before any therapeutic application can be considered.

## Introduction

Focal segmental glomerulosclerosis (FSGS) is a prevalent glomerular condition, accounting for approximately 40% of nephrotic syndrome cases in adults and 20% in children. It represents a significant contributor to steroid-resistant nephrotic syndrome and end-stage renal disease (ESRD) [1].

Although FSGS arises from heterogeneous etiologies, podocyte injury is considered a central and convergent mechanism in disease development. Podocytes are terminally differentiated epithelial cells located on the outer aspect of the glomerular filtration barrier and exhibit limited proliferative capacity under physiological conditions. Following injury, podocytes undergo structural and functional changes, including foot process effacement. Persistent injury may subsequently result in detachment from the glomerular basement membrane and apoptosis, contributing to a progressive reduction in podocyte number and integrity [2].

The clinical presentation of FSGS typically includes proteinuria and can be accompanied by nephrotic syndrome, microscopic hematuria, hypertension, or tubular dysfunction. Complications associated with this disorder include increased risk of infections and deep vein thrombosis [3]. Prognostically, FSGS is unfavorable, with approximately half of patients advancing to ESRD within 5–10 years, representing nearly 20% of dialysis-dependent individuals [4].Currently, definitive diagnosis relies heavily on invasive renal biopsy. This procedure is limited by sampling errors, inability to monitor disease dynamics in real-time, and potential procedural risks [5]. Therapeutic strategies primarily include non-specific immunosuppression and supportive care. However, a significant proportion of patients exhibit treatment resistance or frequent relapses. These challenges highlight the urgent need for reliable tissue-derived molecular biomarkers and novel therapeutic targets [6]. With rapid advancements in transcriptomics, bioinformatic analyses based on gene expression profiles have become widely used to explore characteristic biomarkers [7,8]. The Gene Expression Omnibus (GEO) database from the National Center for Biotechnology Information (NCBI) provides comprehensive gene expression profiles for various diseases, enabling extensive data analyses. Zhang et al. analyzed GEO datasets and identified TGFB1 and NOTCH1 as potential key biomarkers closely associated with glomerular pathogenesis in FSGS, emphasizing the critical role of T-cell infiltration in disease progression [9]. Ge et al. applied an integrative approach using nine distinct machine learning algorithms, combining clinical pathology data to develop a diagnostic model with high precision. Their model identified CD99, OAZ2, and NK-p46 as pivotal biomarkers of FSGS, underscoring the essential role of natural killer cells in the underlying disease pathology [5]. Thus, diverse bioinformatic methodologies provide valuable avenues for discovering potential biomarkers and therapeutic strategies.

WGCNA is a systems biology methodology employed to analyze gene correlation patterns in microarray data [10,11]. It identifies significantly dysregulated genes, clusters these into co-expression modules based on strong correlations, and highlights modules associated with clinical traits, aiding in the identification of novel biomarkers for diagnostics and therapy [12]. SVM-RFE is a supervised machine learning technique that iteratively removes the least informative features to select a minimal optimal gene subset [13]. RF, another machine learning technique based on decision trees, evaluates variable importance by scoring contributions to predictive models [14]. Dysregulated immune cell populations significantly contribute to various disease mechanisms. Substantial evidence suggests that immunological alterations, particularly pro-inflammatory or pro-fibrotic microenvironments, are closely associated with FSGS progression and glomerular scarring, ultimately leading to ESRD [15]. Therefore, investigating ICI in FSGS is crucial for understanding disease pathogenesis and progression.

Gholaminejad et al. [16]employed integrative bioinformatic approaches to investigate FSGS pathogenesis. However, their study did not incorporate machine learning methods or immune cell analyses. In the current study, we included these approaches to identify potential key biomarkers for FSGS and elucidate underlying disease mechanisms.

In the current study, WGCNA was used to establish a co-expression network from a training dataset, leading to the identification of modules significantly linked to FSGS. Subsequently, candidate genes were screened using RF, SVM-RFE, and the cytoHubba MCC algorithm. Gene set enrichment analysis (GSEA) was employed to explore biological processes and signaling pathways related to these central genes. ROC curves were generated to evaluate the diagnostic effectiveness of identified hub genes. ICI analyses were conducted using CIBERSORT to examine relationships between identified genes and immune cell profiles. An external dataset was used to validate these findings in data from another institution and platform.

## Materials and methods

### Data collection and preprocessing

Data were obtained from the GEO database, including the training dataset GSE104948 and the validation dataset GSE129973. The dataset GSE104948 comprised 37 samples (16 FSGS glomerular samples and 21 control samples), profiled on the GPL22945 platform. The external dataset GSE129973 consisted of 40 samples (20 FSGS glomerular samples and 20 control samples), profiled on the HTA 2.0 platform (GPL17586). In addition, a list of differentially expressed genes was obtained using the GEO2R tool. The training and validation cohorts were derived from different patient populations (GSE104948, European Renal cDNA Bank; GSE129973, Nanjing University), and no patient overlap existed between them. Among these 16 samples, 10 had histologically confirmed FSGS and 6 presented overlapping FSGS/MCD features, as annotated in the original European Renal cDNA Bank (ERCB) series; the remaining samples in the series (diabetic nephropathy, pure MCD, and RPGN) were not included in the analysis. Detailed sample information, including GEO accession identifiers, is provided in S1 Table. Quality control of both cohorts comprised inspection of per-sample expression distributions, principal component analysis and hierarchical clustering, and no sample was excluded (S1 Fig and S2 Fig; per-sample statistics in S3 Table).

### WGCNA

A gene co-expression network was constructed using the WGCNA package integrated within TBtools-II [17]. The depositor-provided, log2-scale gene-level expression matrix (annotated using the Human Entrez Gene ID custom CDF, version 19) comprising 12,074 Entrez-level identifiers was used as input; during annotation to official gene symbols, 29 identifiers lacking a one-to-one current official gene symbol (retired or merged Entrez IDs) were removed, leaving 12,045 unique gene-level entries, from which the 8,000 genes with the highest median absolute deviation (MAD) were retained to establish a scale-free network, using the default block size (5,000 genes) of the WGCNA plugin. A soft-thresholding power of 8 (R^2^ = 0.85) was determined appropriate. Module detection parameters were configured with a minimum module size of 30, and cutree height at 0.25. Modules demonstrating high similarity were merged. Genes within clinically relevant modules with absolute MM and GS values of at least 0.5 were considered candidate genes and selected for subsequent analyses. The complete pipeline, including the module-detection settings, is provided in the S9 Dataset and regenerates the Fig 1 panels.

**Fig 1:**
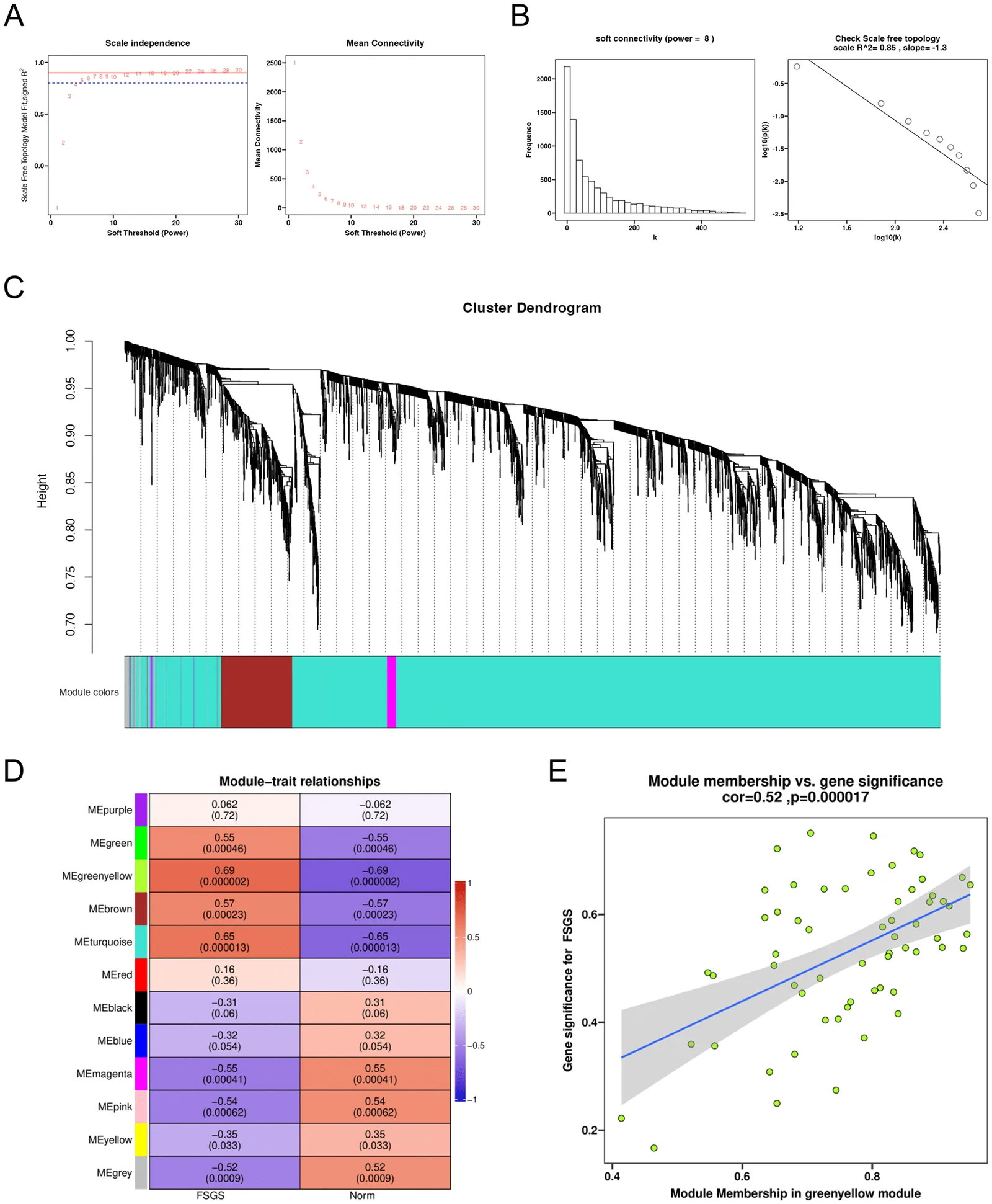
WGCNA of the training cohort. (A) Selection of the soft-thresholding power: scale independence (left) and mean connectivity (right). (B) Distribution of network connectivity at the selected power (β = 8) and verification of the scale-free topology fit. (C) Gene clustering dendrogram with co-expression modules labelled by colours. (D) Module-trait relationship heatmap showing the Pearson correlation between module eigengenes and clinical status (FSGS or control). (E) Scatter plot of module membership versus gene significance for FSGS in the greenyellow module. Statistics: module-trait associations were evaluated by Pearson correlation; the soft-thresholding power was selected as the lowest power satisfying the scale-free topology criterion.

### Screening of core genes

To refine the list of candidate genes, feature selection was implemented using RF, SVM-RFE, and the cytoHubba MCC algorithm. The RF classifier generated multiple decision trees, and feature importance was evaluated using the Mean Decrease Gini (MDG) index [14]. The random forest model used 500 decision trees, and genes with MDG > 1.0 were identified as candidate hub genes [18]. SVM-RFE ranked the candidate genes by recursively eliminating the feature carrying the smallest squared weight and used five-fold cross-validation of the training cohort [13]. Specifically, SVM-RFE was re-implemented as a self-contained script in R with the e1071 package (linear kernel, cost = 1, stratified five-fold cross-validation with a fixed fold assignment), and the five top-ranked features were retained; random forest analyses were performed with the randomForest package, and the results were visualized separately. All preprocessing, gene filtering, WGCNA network construction and feature selection procedures (RF, SVM-RFE, and cytoHubba MCC) were performed exclusively on the training cohort to avoid information leakage; the independent validation cohort was processed separately and was used only for evaluation. To quantify the selection stability across folds of the supervised selection steps, random forest (500 trees) and linear SVM-RFE were additionally re-run independently inside the training portion of 50 outer folds (10 repeats of five-fold stratified cross-validation of the 37 training samples; scikit-learn 1.9.0), and per-gene selection frequencies were recorded (S7 Table). Because Gini importance magnitudes scale with cohort size, the MDG > 1.0 threshold is not fold-portable (it was met in 0 of 50 training folds); the fold-wise analysis therefore used a fixed top-5 rule mirroring the five-gene random-forest subset. The cytoHubba MCC step was not repeated fold-wise because it depends only on the protein-interaction topology of the candidate list and not on the samples.

The PPI network was generated through the STRING database using a minimum required interaction score = 0.4 (medium confidence) and visualized in Cytoscape. To identify key genes within the network, the MCC algorithm in the cytoHubba plugin was applied. The 20 genes with the highest MCC scores were selected for subsequent analyses and visualization [19]. Hub genes were identified by intersecting the results obtained from the three analytical methods. The analysis was performed using R software, and visualization was conducted using ggplot2 (v3.4.4) and VennDiagram (v1.7.3).

### Functional enrichment analyses

Functional enrichment, comprising Gene Ontology (GO) and Reactome pathway analyses, was performed on the 39 candidate genes identified from the greenyellow module (36 of which are annotated in GO and 26 in Reactome). Functional enrichment of GO terms was carried out using the clusterProfiler package in R, whereas Reactome pathway analysis was conducted with ReactomePA. Visualization of the results was performed with ggplot2. Terms with an adjusted p-value (FDR) < 0.05 were considered significantly enriched.

GSEA was performed using the clusterProfiler package based on the Canonical Pathways (CP) subcollection of the C2 curated gene sets collection in the Molecular Signatures Database (MSigDB). Genes were ranked according to log2 fold change values between FSGS and control samples. Pathways with adjusted p-value < 0.05 were considered statistically significant. The enriched gene sets were grouped into two functional programs, and four representative gene sets from each program are shown.

### Diagnostic evaluation and immune infiltration analysis

ROC curves were used to assess the diagnostic capabilities of core genes. The area under the ROC curve (AUC) and corresponding 95% confidence intervals were calculated; AUC is threshold-free and was used as the primary performance metric. Descriptive operating points (sensitivity, specificity, overall accuracy) were derived at the Youden-optimal cut-off (the threshold maximizing sensitivity + specificity) within a single cohort. Because the training and validation cohorts were profiled on different platforms with non-overlapping log2 intensity scales, a cut-off calibrated in one cohort cannot be transferred to the other, and no threshold-based classification metrics are reported for the external validation cohort. ROC curve analysis was conducted using the pROC package, and the results were visualized using the ggplot2 package. ICI levels were estimated to investigate associations between the identified core genes and the IME in FSGS. Immune infiltration was quantified using CIBERSORT (v1.03) with the LM22 reference matrix to infer the relative proportions of 22 immune cell types from bulk gene expression data. CIBERSORT was run on the full deposited series (71 samples, including the 37 training samples; 1,000 permutations, quantile normalization enabled), and only the training samples were retained for downstream analysis; among the 37 training samples, 12 had a CIBERSORT deconvolution P value < 0.05 (8 FSGS: GSM2810777, GSM2810779, GSM2810780, GSM2810784, GSM2810787, GSM2810789, GSM2810790, GSM2810791; 4 controls: GSM2810798, GSM2810801, GSM2810806, GSM2810839), and per-sample significance values are provided in S5 Table. Group comparisons of the inferred cell fractions were performed on the full training cohort (n = 37; primary analysis) and, descriptively, on the 12-sample significance-filtered subset; Benjamini–Hochberg correction was applied across the 20 detectable cell types (two types were zero in all samples). As an internal check of the deconvolution, the CIBERSORT fractions were compared with the mean expression of canonical marker genes measured independently of CIBERSORT (monocytes: CD14, LST1, S100A8, S100A9, FCN1; activated NK cells: NKG7, NCR1, KLRF1, GNLY, PRF1; naive B cells: MS4A1, CD79A, BANK1, CD19; CD8 T cells: CD8A, CD8B, GZMK; S8 Table). To relate the inferred immune landscape to the two hub genes, we constructed a pairwise correlation matrix across the inferred immune cell subsets using Pearson’s correlation, whereas the associations between the expression of each hub gene and each immune cell subset were assessed using Spearman’s rank correlation, as the estimated cell fractions are bounded proportions for which a linear relationship with gene expression cannot be assumed. Gene–cell correlations were computed on all 37 training samples across the 20 detectable cell types, with Benjamini–Hochberg correction across the 40 tests; all raw and adjusted P values are provided in S8 Table. In the correlation heatmap, block size in the cell–cell matrix and link width encode the absolute correlation strength, and link color encodes the corresponding P value in three classes (<0.01, 0.01–0.05 and ≥0.05).

### External validation

Differential expression analysis and ROC curve analysis were performed in an independent external validation dataset (GSE129973; 20 FSGS vs 20 unaffected controls, glomerular compartment). No patient overlap existed between the training and validation cohorts.

### Statistical analysis

All statistical analyses and data visualizations were carried out using R software (version 4.2.1). For the differential-expression and functional-enrichment analyses, P values were adjusted for multiple testing using the Benjamini–Hochberg procedure. For the immune-infiltration analysis, cell–cell associations were computed using Pearson’s correlation and gene–cell associations using Spearman’s rank correlation, and the resulting P values were grouped into three significance classes for visualization. These analyses quantify association only and do not establish a regulatory relationship. Two-group comparisons (FSGS vs control) were performed using the Wilcoxon rank-sum test. Statistical significance was defined as P < 0.05, categorized as follows: P < 0.05 (*), P < 0.01 (**), P < 0.001 (***), and P < 0.0001 (****), with “ns” denoting no statistical significance. All analyses were performed in R 4.2.1 (WGCNA via TBtools-II v2.439; e1071; randomForest; clusterProfiler; ReactomePA; pROC; ggplot2) and Python 3.13 (numpy 2.5.1; scipy 1.18.0; scikit-learn 1.9.0); a complete software and version list is provided in the deposited analysis pipeline (S9 Dataset).

## Results

### WGCNA results

After preprocessing, 12,074 Entrez-level identifiers were annotated to 12,045 unique gene-level entries, and the 8,000 genes with the highest median absolute deviation were used to construct the WGCNA network. A soft-thresholding power of β = 8 (R2 = 0.85) was selected based on the calculated scale-free topology fit index (Fig 1A-1B). Subsequently, a gene clustering dendrogram was constructed (Fig 1C). Hierarchical clustering, using the topological overlap matrix (TOM), resulted in the merging of similar modules (Fig 1D), ultimately forming 12 distinct co-expression modules. Correlation analysis between modules and clinical traits indicated that the greenyellow module exhibited the strongest association with FSGS (r = 0.69, p = 2 × 10^-6^). Fig 1E illustrates the correlation between MM and GS within the identified module. A total of 39 candidate genes, with absolute MM and GS values of at least 0.5, were designated as potential key genes related to FSGS (S2 Table).

### Enrichment analysis results

#### GO and reactome enrichment analysis.

GO enrichment analyses indicated that identified genes primarily participate in processes related to cell-cycle regulation and mitosis, particularly the positive modulation of cell-cycle activities and regulation of mitotic phase progression (Fig 2A). Reactome pathway enrichment further demonstrated significant involvement of AURKA in APC/C-mediated proteolysis of cell-cycle proteins and mitotic cycle control (-log10 p-value > 5.75); CENPM exhibited enrichment in processes such as resolution of sister chromatid cohesion and mitotic spindle checkpoint, with maximal enrichment noted in mitotic anaphase and metaphase/anaphase phases (-log10 p-value > 6) (Fig 2B). Collectively, these results suggest that central genes predominantly influence mitotic regulation and cell-cycle progression, highlighting abnormal mitotic activation as a crucial biological aspect of FSGS.

**Fig 2:**
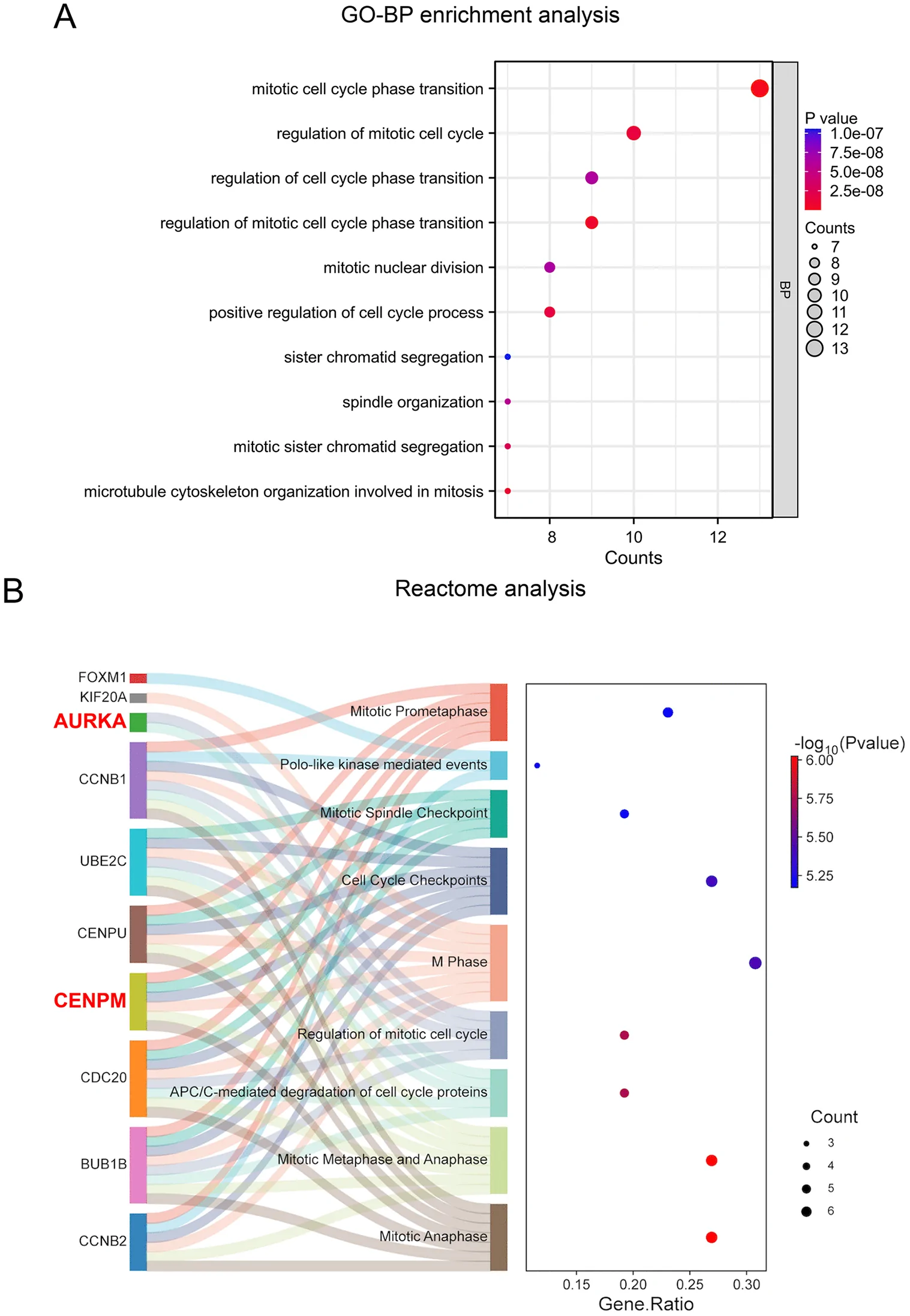
Functional enrichment of the candidate genes. (A) GO-BP enrichment analysis: bubble plot of the ten most significantly enriched Gene Ontology biological-process (GO-BP) terms; dot size and color encode the gene count and nominal P value, respectively (significance was assessed on Benjamini–Hochberg-adjusted P values). (B) Reactome analysis: a Sankey diagram linking the candidate genes to their enriched pathways (left) and the corresponding bubble plot of gene ratio and counts (right). Statistics: enrichment analysis was performed with clusterProfiler and ReactomePA; p-values were adjusted for multiple testing using the Benjamini–Hochberg procedure, and terms with adjusted p < 0.05 were considered significant.

### Selection of hub genes

Feature selection was conducted using two machine learning methods: RF and SVM-RFE. In RF analysis, genes with a Mean Decrease Gini (MDG) > 1 were considered significant, resulting in 5 candidate genes (Fig 3A). In SVM-RFE analysis, the five top-ranked features (AURKA, CSHL1, CCNB1, FN1 and CENPM) were retained (Fig 3B); each of them was also recovered within the top 12 of at least four of the five cross-validation folds. Moreover, a PPI network was developed using the STRING database. Core genes were identified through cytoHubba’s MCC algorithm, selecting the top 20 genes for network visualization via Cytoscape (Fig 3C). Final hub genes were confirmed by intersecting outcomes from multiple methodological analyses (Fig 3D). The intersection remained unchanged (AURKA and CENPM) across all 24 combinations of random-forest random seed and MDG threshold that were tested (S6 Table). A fold-wise stability analysis (feature selection re-run independently inside the training portion of 50 outer folds; S7 Table) showed that AURKA was recovered among the top five genes in 38/50 folds by random forest and 47/50 folds by SVM-RFE (both methods: 36/50 folds), whereas CENPM was recovered less consistently (33/50, 4/50 and 4/50, respectively). AURKA is therefore the more robustly selected of the two candidates, and CENPM’s selection is partly configuration-dependent at this sample size.

**Fig 3:**
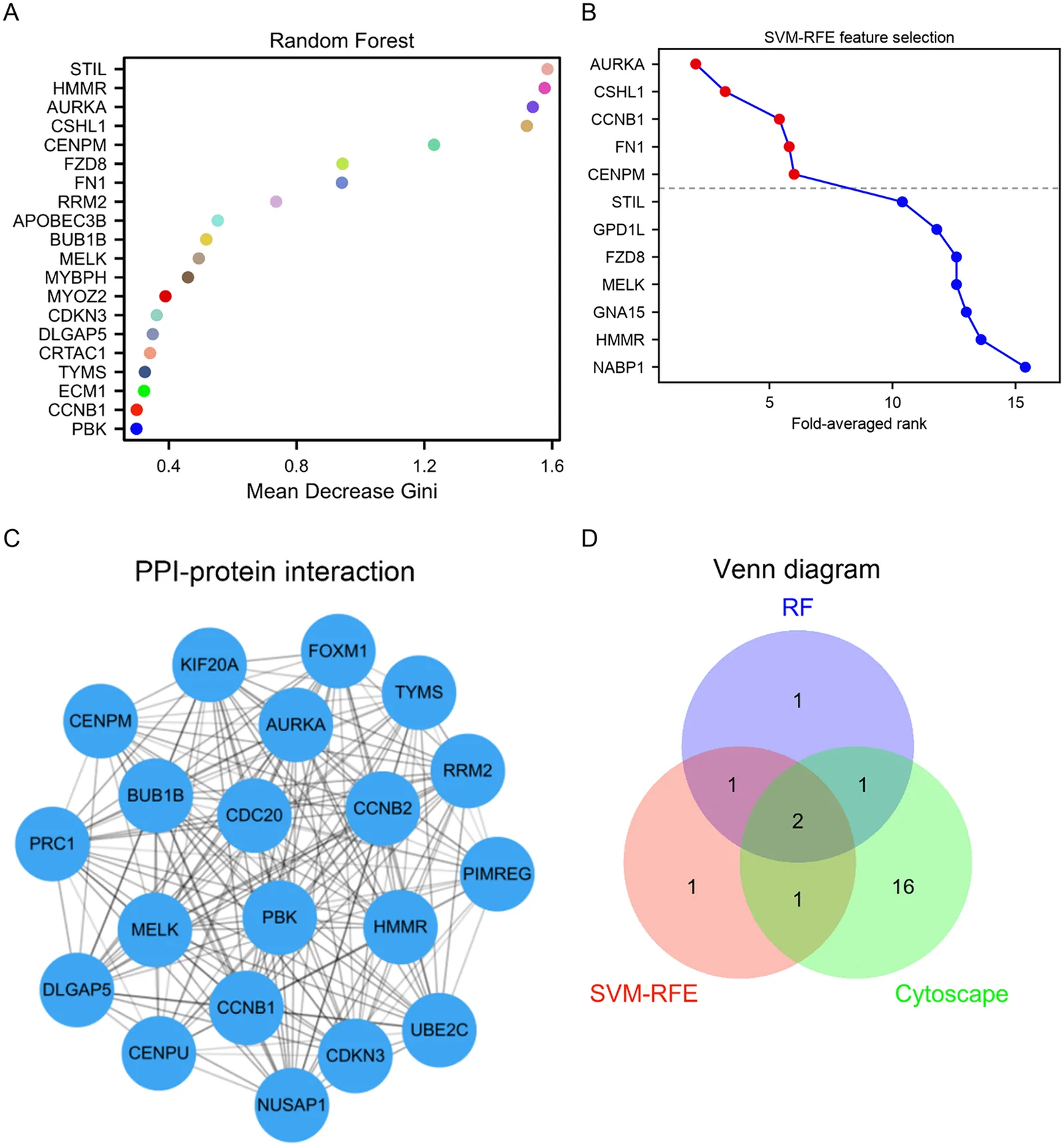
Hub gene selection. (A) Random-forest feature importance ranked by Mean Decrease Gini; genes above the MDG > 1 selection cut-off were retained as candidates, and they are separated from the remaining genes by a marked gap in importance. (B) SVM-RFE feature selection: the 12 top-ranked candidate genes plotted in order of fold-averaged elimination rank (rank 1 = most important) and connected by a solid line; the five retained features are shown in red and the retention cut-off is marked by the dashed line. (C) Protein–protein interaction network of the candidate genes (STRING, medium confidence) visualized in Cytoscape, showing the top 20 MCC-ranked genes as nodes. (D) Venn diagram of the genes selected by RF (five genes), SVM-RFE (five genes) and cytoHubba MCC (top 20), with the intersection highlighted. Statistics: all feature-selection procedures were performed on the training cohort only; the random-forest model used 500 trees, and the SVM-RFE run used a fixed fold assignment and is reproducible from the deposited analysis pipeline (S9 Dataset).

### GSEA

GSEA elucidated potential pathogenic mechanisms in FSGS. Genes ranked by differential expression between FSGS and control samples were enriched in pathways involved in protein translation, APC/C-mediated degradation of cell cycle regulators, APC/C–CDH1-dependent turnover of CDC20 and its substrates during late mitosis and early G1 phase, as well as mitochondrial translation (Fig 4A); AURKA itself is a leading-edge (core-enriched) gene of the two APC/C gene sets. A second group of enriched pathways comprised M phase-related processes, including metaphase–anaphase transition, sister chromatid separation, and cell cycle checkpoint regulation (Fig 4B), and CENPM is a leading-edge gene of all four of these gene sets. These findings indicate that AURKA is more closely associated with processes governing cell cycle regulation and metabolic activity during mitotic exit and early G1 progression, whereas CENPM primarily reflects active mitotic progression and chromosomal segregation. The results suggest heterogeneous cell cycle dysregulation in FSGS, characterized by regulatory alterations associated with AURKA and mitotic structural activity linked to CENPM.

**Fig 4:**
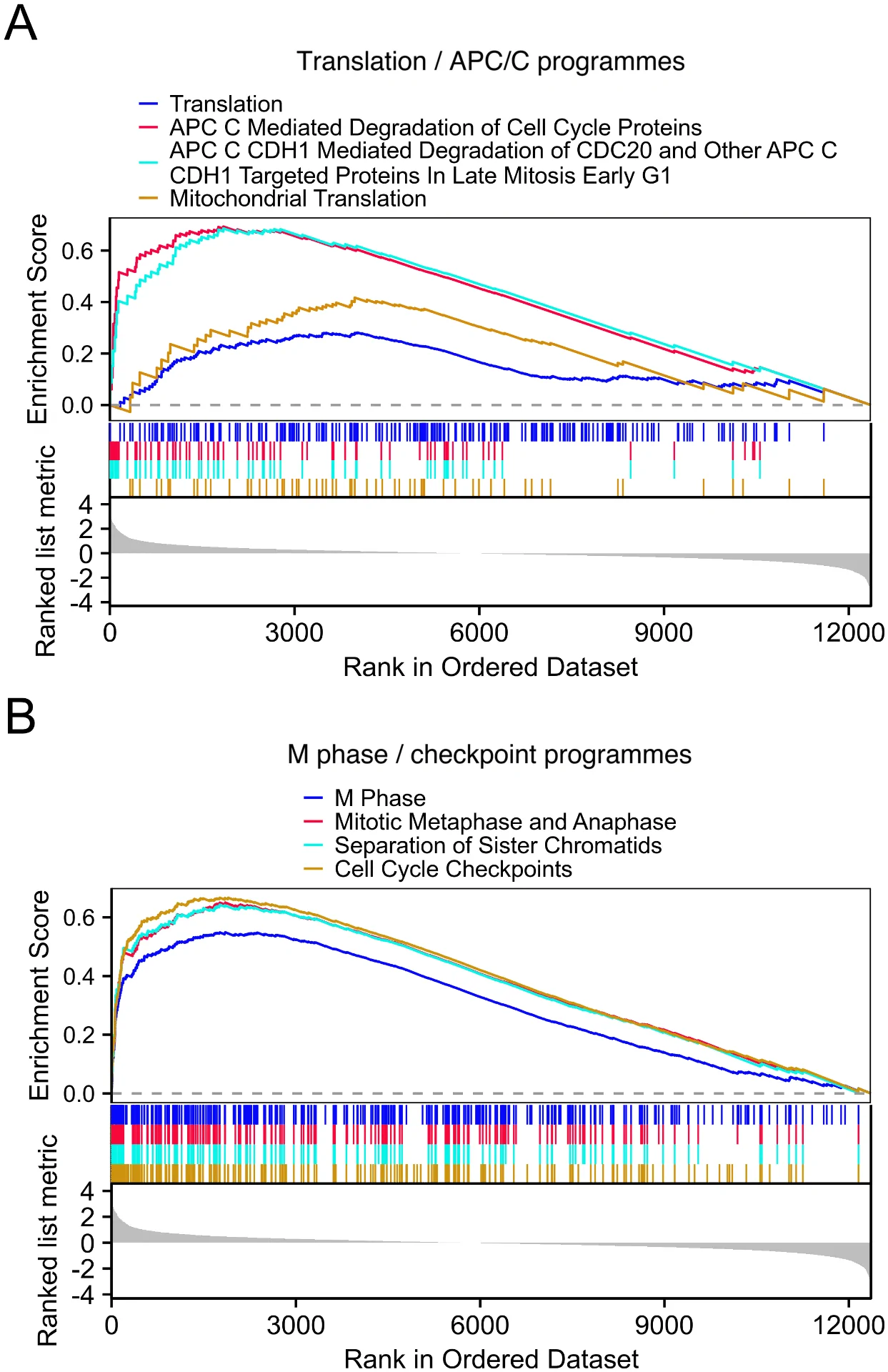
Gene set enrichment analysis of the FSGS versus control comparison. (A) Enrichment plots for translation- and APC/C-related gene sets; AURKA is a leading-edge (core-enriched) gene of the APC/C gene sets. (B) Enrichment plots for M phase, sister-chromatid segregation and checkpoint gene sets; CENPM is a leading-edge gene of all four gene sets. In each panel the running enrichment score (top) and the ranked-list metric (bottom) are shown. Statistics: GSEA was performed with clusterProfiler against the curated pathway (CP) subcollection of MSigDB C2; genes were ranked by log2 fold change, and pathways with adjusted p < 0.05 were considered significant.

### ROC analysis

Box plots were used to illustrate expression differences of hub genes, while ROC curves evaluated their diagnostic performance. AURKA expression was higher in the FSGS group than in controls (log2FC = 0.47, p < 0.001; AUC = 0.95) (Fig 5A(i), 5B(i)). Similarly, CENPM was upregulated in the FSGS group (log2FC = 0.40, p < 0.001; AUC = 0.94) (Fig 5A(ii), 5B(ii)). These results indicate that both AURKA and CENPM show high discriminative performance in the training cohort, in contrast to the lower performance in the independent validation cohort.

**Fig 5:**
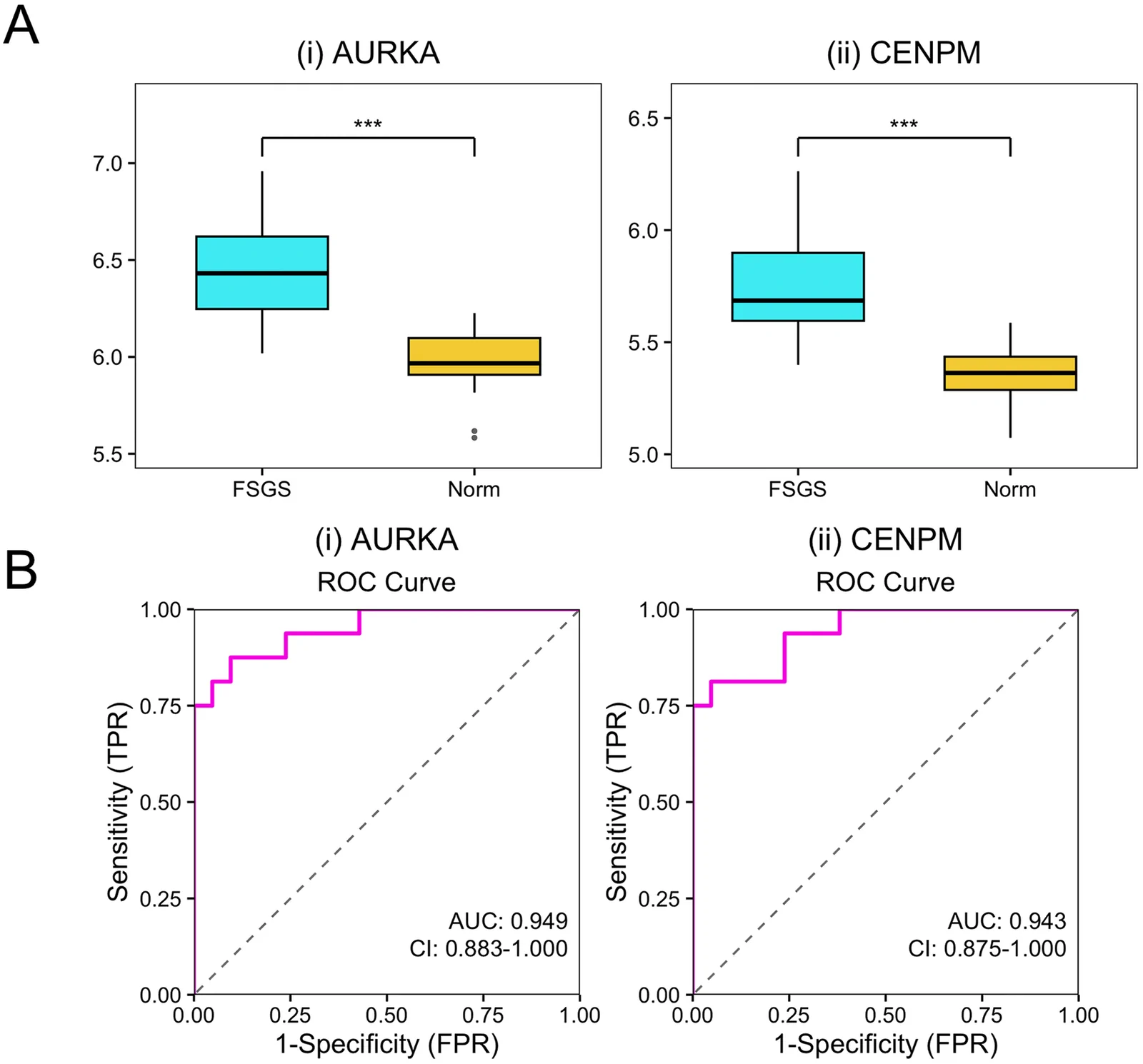
Expression and ROC analysis of AURKA and CENPM in the training cohort (16 FSGS vs 21 controls). (A) Box plots of log2-transformed expression values for AURKA (i) and CENPM (ii), colored by group. (B) ROC curves for AURKA (i) and CENPM (ii), with the grey diagonal marking the reference line. Statistics: two-group comparisons were performed with Wilcoxon rank-sum tests; AUC and 95% confidence intervals were computed with the pROC package.

### ICI analysis results

Fig 6A shows the inferred immune landscape. In the full training cohort (n = 37), monocytes (Benjamini–Hochberg-adjusted P = 7.9 × 10^-5^) and activated NK cells (adjusted P = 0.007) were significantly more abundant in FSGS, whereas naive B cells (adjusted P = 0.002), neutrophils (adjusted P = 0.008) and resting and activated dendritic cells (adjusted P = 0.027 for both) were significantly less abundant after correction across the 20 detectable cell types, suggesting an altered immune infiltration landscape in FSGS. Within the 12-sample subset with significant deconvolution (8 FSGS vs 4 controls), the same directional differences were observed but no comparison survived correction (smallest adjusted P = 0.051), as expected at this sample size; subset-level comparisons are therefore descriptive only (S8 Table). As an internal check, the CIBERSORT monocyte and activated NK cell fractions correlated with the mean expression of their canonical marker genes (Spearman rho = 0.60, adjusted P = 3.5 × 10^-4^, and rho = 0.48, adjusted P = 0.005, respectively), whereas naive B cell and CD8 T cell estimates did not (rho = 0.08 and −0.17, n.s.), consistent with the leukocyte origin of the LM22 reference (S8 Table). Notably, CD4+ naive T cells and eosinophils were not detected in the analyzed samples.

**Fig 6:**
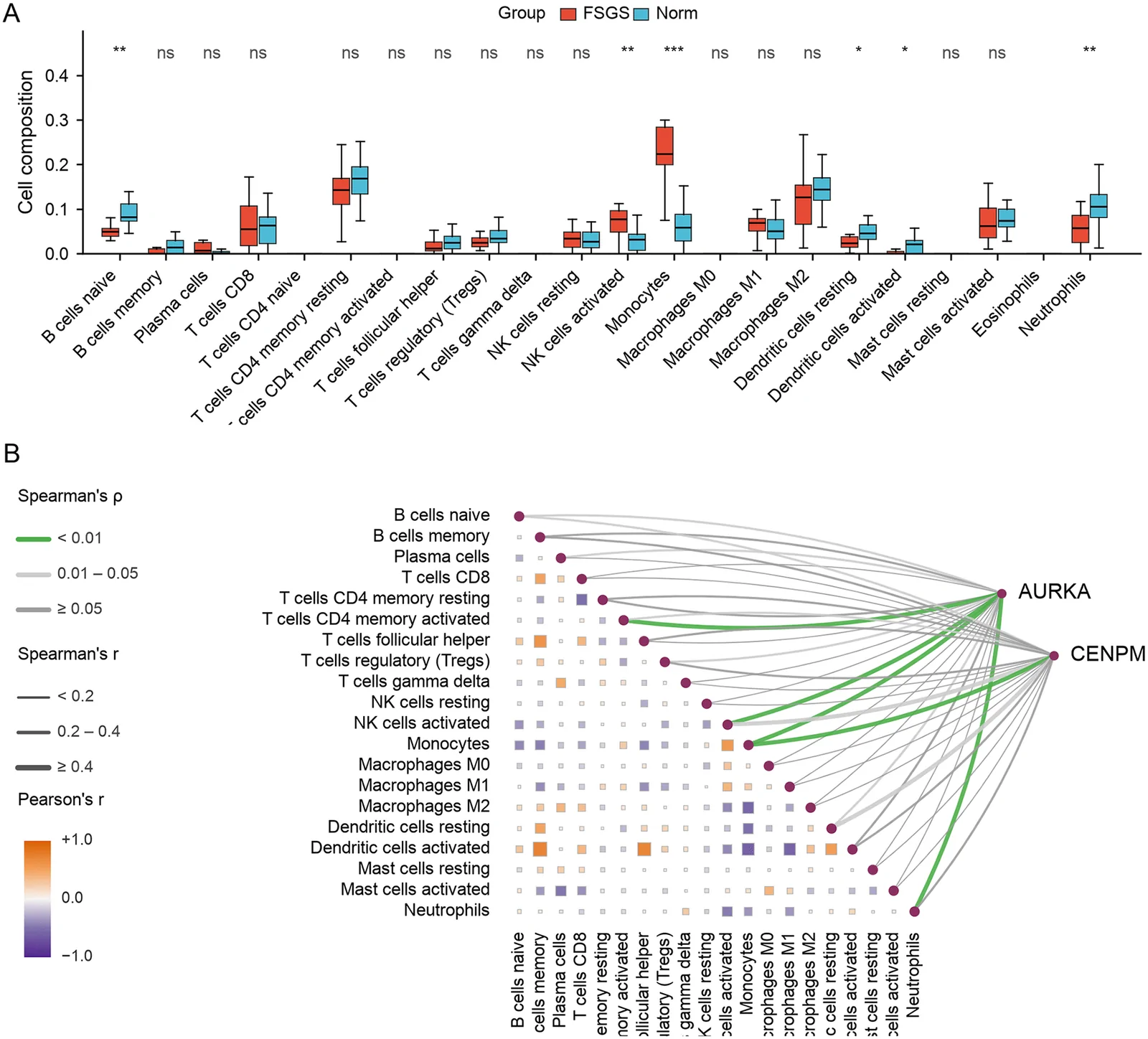
Immune cell infiltration analysis. (A) Relative fractions of immune cell subsets estimated by CIBERSORT (LM22 signature) in FSGS versus normal controls; significance stars above the boxes indicate between-group comparisons. (B) Heatmap of pairwise Pearson correlations among immune cell subsets, overlaid with links connecting AURKA and CENPM to immune cell profiles; link color denotes the significance class of the Spearman’s rank correlation and link width its strength. Statistics: CIBERSORT deconvolution was performed with the LM22 signature matrix (1,000 permutations, quantile normalization); significant deconvolution (P < 0.05) was obtained in 12 of 37 samples (S5 Table); between-group comparisons of cell fractions used Wilcoxon rank-sum tests on the full training cohort (n = 37), with Benjamini–Hochberg correction across the 20 detectable cell types; gene–immune and cell–cell associations were assessed by Spearman’s rank and Pearson correlation analyses, respectively, with Benjamini–Hochberg correction across the 40 gene–cell tests; exact values are provided in S8 Table.

Fig 6B illustrates the Spearman’s rank correlations between hub-gene expression and immune cell populations, computed on all 37 training samples across the 20 detectable cell types. Both genes correlated most strongly with monocytes (CENPM: rho = 0.61; AURKA: rho = 0.58) and activated NK cells (CENPM: rho = 0.40; AURKA: rho = 0.50), and negatively with neutrophils, dendritic cells and several lymphocyte subsets (S8 Table). After Benjamini–Hochberg correction across the 40 gene–cell tests, three associations remained significant: CENPM–monocytes (adjusted P = 0.002), AURKA–monocytes (adjusted P = 0.003) and AURKA–activated NK cells (adjusted P = 0.022). These results suggest that AURKA and CENPM are associated with the immune microenvironment in FSGS, particularly involving monocyte and activated NK cell infiltration; the remaining associations did not survive multiple-testing correction and should be considered exploratory. These associations do not imply causality.

### Validation using an external dataset

Validation using an independent external dataset (GSE129973; 20 FSGS vs 20 controls) corroborated the direction of expression change of the identified hub genes. AURKA was upregulated in FSGS (log2FC = 0.18, adjusted p = 0.012), whereas CENPM showed a weaker change (log2FC = 0.07, adjusted p = 0.22). Wilcoxon rank-sum tests on the same cohort gave P = 0.0013 for AURKA and P = 0.017 for CENPM (Fig 7A(i), 7A(ii)). The adjusted P values reported above were obtained from the genome-wide limma analysis in GEO2R, whereas the Wilcoxon P values were unadjusted. Because the two cohorts were profiled on different platforms (GPL22945 vs HTA 2.0) with non-overlapping log2 intensity scales, no cut-off calibrated on the training cohort can be transferred to the validation cohort; external performance is therefore reported threshold-free as AUC: 0.797 (95% CI 0.661–0.934) for AURKA and 0.720 for CENPM (95% CI 0.553–0.887) (Fig 7B(i), 7B(ii)). For descriptive completeness only, the Youden-optimal operating point of the validation ROC itself — a post-hoc threshold selected on the validation data, carrying no independent interpretation — corresponds to 90.0% sensitivity and 60.0% specificity (overall accuracy 75.0%) for AURKA and 90.0% sensitivity and 55.0% specificity (overall accuracy 72.5%) for CENPM. These findings support AURKA as an exploratory candidate biomarker. For CENPM, the external evidence is weak and internally discordant — a moderate AUC despite a non-significant adjusted expression difference (adjusted p = 0.22) — and should not be interpreted as independent confirmation; CENPM is accordingly presented as the weaker of the two candidates, requiring further validation.

**Fig 7:**
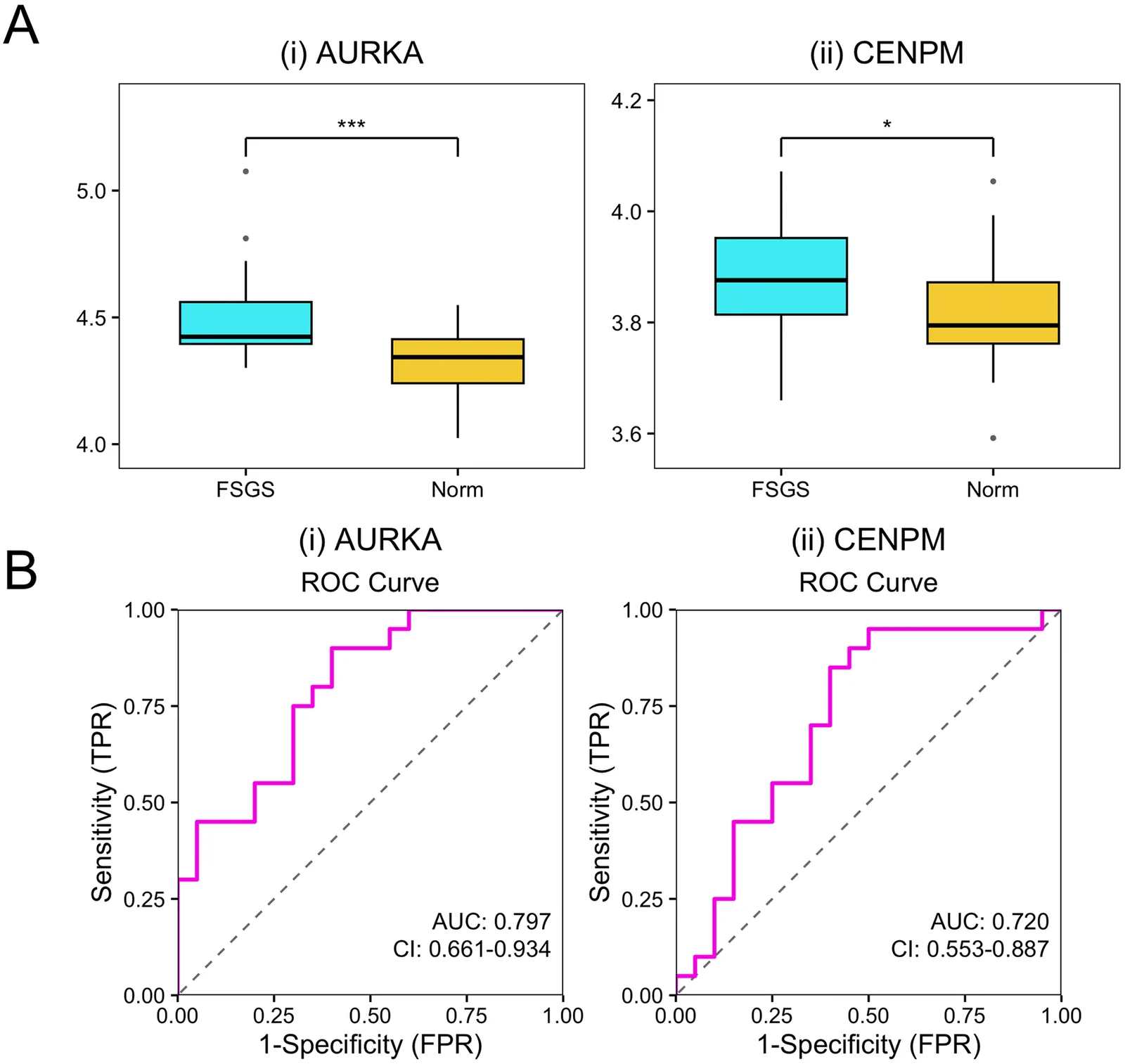
Expression and ROC analysis of AURKA and CENPM in the independent validation cohort (20 FSGS vs 20 controls). (A) Box plots of log2-transformed expression values for AURKA (i) and CENPM (ii), colored by group. (B) ROC curves for AURKA (i) and CENPM (ii), with the grey diagonal marking the reference line. Statistics: two-group comparisons were performed with Wilcoxon rank-sum tests; AUC and 95% confidence intervals were computed with the pROC package.

## Discussion

FSGS is a clinicopathological syndrome characterized by shared histopathological features, primarily diagnosed by renal biopsy. Clinically, FSGS presents several challenges, including variable treatment responses, high recurrence rates, and heterogeneous prognoses [20]. Thus, identifying novel molecular pathways to enhance diagnosis and treatment remains critical.

Through integrated analyses of two GEO datasets employing WGCNA, machine learning methodologies, and PPI networks, this study identified AURKA and CENPM as candidate hub genes. Their discriminative performance was assessed by ROC analysis. GSEA identified biological processes and signaling pathways associated with these genes, and immune correlations were examined through CIBERSORT. Both genes showed expression changes in the same direction in the independent external cohort as in the training cohort, although their AUCs were lower (0.797 for AURKA and 0.720 for CENPM).

AURKA is a serine/threonine kinase involved in essential cellular functions, including cell-cycle initiation, centrosome dynamics, spindle formation, and chromatid segregation [21]. It is integral to the p53/TP53 pathway, particularly in checkpoint responses implicated in oncogenesis, and functions primarily through p53 phosphorylation and destabilization [22]. Aurora kinases are frequently overexpressed in malignancies. Aberrant activation of AURKA due to excessive formation of the AURKA/TPX2 complex promotes mitotic abnormalities, aneuploidy, and chromosomal instability. Targeting this complex may therefore represent a promising therapeutic strategy for cancer treatment [23]. Zhou et al. reported that AURKA may serve as a hub gene involved in the comorbidity of rheumatoid arthritis and major depressive disorder, with aucubin (AU) exerting therapeutic effects by downregulating CXCL10 and BTN3A2 in PC12 cells [24]. In renal research, Jiang et al. demonstrated that AURKA activates the TGF-β1/Smad pathway, thereby promoting renal fibroblast proliferation and activation. This activation upregulates fibrotic markers, including α-SMA, fibronectin, and collagen I, leading to increased extracellular matrix deposition and renal interstitial fibrosis. Treatment with MK-5108 significantly inhibited AURKA activity, reduced collagen deposition and profibrotic factor expression, and alleviated renal pathology. These findings suggest that AURKA exerts profibrotic effects in chronic kidney disease (CKD) and that targeted inhibition may delay CKD progression and renal fibrosis [25]. In addition, previous studies have documented abnormal expression and activation of AURKA in renal carcinoma and renal cysts associated with polycystic kidney disease (PKD) [26–28]. In the current analysis, AURKA was moderately upregulated in the FSGS cohort and was prominently involved in cell-cycle regulation. AURKA expression was positively correlated with the estimated fractions of monocytes and activated NK cells after correction for multiple testing. The negative correlation with the estimated fraction of resting memory CD4 T cells did not remain significant after correction. These observations are consistent with previous studies and indicate the need for further investigation.

CENPM, also known as PANE1 or C22orf18, encodes a centromeric protein that is required for kinetochore assembly and plays an important role in chromosome segregation during mitosis [29]. Dysregulated CENPM expression has been reported in multiple malignancies, in which increased expression facilitates tumor progression and is associated with worse patient outcomes [30,31]. CENPM is involved in chromosome segregation during cell division and may contribute to tumor cell proliferation and invasion [32,33]. Furthermore, Zhang et al. reported a positive association between CENPM expression and the infiltration of various immune cell populations, including CD8+ T lymphocytes, regulatory T cells (Tregs), macrophages, and dendritic cells. High CENPM expression promotes an immunosuppressive tumor microenvironment, enhances immune evasion, and is associated with increased expression of immune checkpoint molecules, thereby affecting prognosis [34]. To our knowledge, this is the first study to suggest a potential role for CENPM in FSGS.

The GSEA results revealed distinct but interconnected functional programs within the FSGS signature. One group of enriched pathways, whose APC/C members contain AURKA in their leading edge, related to protein translation, APC/C-CDH1-mediated degradation of cell-cycle regulators, and mitochondrial translation, suggesting dysregulated mitotic exit, early G1 re-entry, and altered metabolic activity. The second group, all of whose members contain CENPM in their leading edge, involved M phase progression, sister chromatid separation, and cell-cycle checkpoint regulation, indicating mitotic stress and aberrant chromosomal segregation. These findings suggest a layered pattern of cell-cycle dysregulation in FSGS, in which AURKA may be involved in upstream cell-cycle reprogramming and metabolic adaptation, whereas CENPM may reflect downstream defects in mitotic execution. Such aberrant activation of the cell-cycle machinery may contribute to renal cell dysfunction and disease progression.

Previous studies have shown that podocyte injury can induce inappropriate cell-cycle re-entry, often resulting in failed mitotic completion, mitotic catastrophe, podocyte depletion, and subsequent progressive glomerulosclerosis [35]. Podocyte injury in FSGS has also been closely linked to aberrant cell-cycle re-entry. In particular, the ATM-MAD2B-APC/C signaling axis has been shown to disrupt APC/C activity, leading to failed cell-cycle exit and mitotic catastrophe in podocytes. ATM-mediated stabilization of MAD2L2 inhibits APC/C function, thereby promoting cell-cycle dysregulation and podocyte injury [36]. In our GSEA, AURKA was among the leading-edge genes in APC/C-related gene sets, whereas CENPM was among the leading-edge genes in gene sets related to M phase, sister chromatid segregation, and mitotic checkpoints. The enrichment of APC/C-CDH1-mediated degradation and CDC20 regulation, with AURKA among the leading-edge genes, suggests dysregulated mitotic exit and early G1 re-entry, accompanied by enhanced translational and mitochondrial activity. In contrast, the enrichment of M phase, sister-chromatid segregation and checkpoint pathways, with CENPM among the leading-edge genes, may reflect aberrant mitotic progression and chromosomal segregation stress, indicating downstream defects in mitotic execution. We emphasise that this layered model is a hypothesis generated by enrichment and network analyses; whether AURKA or CENPM functionally regulate these programs in FSGS glomeruli remains to be tested experimentally.

In this study, significantly elevated infiltration of monocytes and activated NK cells was observed in FSGS samples compared with controls. It is well established that monocytes infiltrate injured renal tissues and differentiate into macrophages. These macrophages release pro-inflammatory and pro-fibrotic cytokines, including TNF-α, IL-1β, and TGF-β, thereby promoting persistent inflammation, extracellular matrix accumulation, and renal fibrosis [15,37]. Thus, the increased monocyte infiltration identified in this study likely reflects an inflammatory microenvironment that accelerates disease progression.

Furthermore, Spearman’s rank correlation analysis indicated significant associations between hub genes and immune cell infiltration. Both AURKA and CENPM were positively correlated with monocyte abundance, suggesting their involvement in remodeling the immune microenvironment in FSGS. Previous studies have demonstrated the role of AURKA in inflammatory and fibrotic responses, whereas CENPM has been closely associated with immune regulation and immune cell infiltration in cancers [34]. Consistent with these findings, our results suggest that elevated AURKA and CENPM expression is closely related to immune processes in FSGS. Because these relationships were derived from correlation analysis of bulk tissue, they are associative in nature and cannot distinguish whether the observed immune-cell shifts drive, or arise from, cell-cycle dysregulation.

These immune-related findings should be interpreted with two caveats. First, CIBERSORT estimates relative cell fractions from bulk tissue expression, so the observed differences may partly reflect a shift in tissue cell composition rather than a change in the per-cell expression of AURKA and CENPM; because both genes are expressed at low levels in quiescent glomeruli, a small number of infiltrating cells could disproportionately influence their apparent correlation with immune cell fractions. Second, the LM22 signature matrix was derived mainly from peripheral blood leukocytes [38] and therefore does not fully represent the immune cell populations of the kidney; glomerular tissue contains relatively few resident leukocytes, and several cell types that are prominent in blood are inferred from a limited number of marker genes. The relative differences reported here remain internally comparable because all samples were processed with an identical pipeline and the same signature matrix. Establishing that AURKA and CENPM actually regulate the pathways or immune profiles with which they correlate will require independent experimental work, including detection of the two proteins in situ in FSGS biopsies, cell-type-resolved profiling, and functional experiments in podocytes or glomerular endothelial cells.

Collectively, these findings are consistent with the classical ATM-MAD2B-APC/C framework and suggest a layered model of cell-cycle dysregulation in FSGS, in which AURKA may act upstream to modulate cell-cycle reprogramming and metabolic adaptation, whereas CENPM may reflect downstream mitotic stress. In addition, the observed immune cell infiltration patterns suggest that cell-cycle dysregulation may be coupled with remodeling of the immune microenvironment during disease progression.

Compared with previously reported FSGS biomarkers, the two genes identified here differ in both sample source and intended use. Circulating and urinary candidates, for example soluble urokinase-type plasminogen activator receptor (suPAR) [39,40], anti-nephrin autoantibodies [41,42], and urinary markers such as NGAL and KIM-1 [43], have the advantage of being measurable non-invasively, but their specificity for FSGS, particularly in distinguishing FSGS from other proteinuric glomerulopathies, remains a matter of debate [40,42,44]. AURKA and CENPM were derived from glomerular tissue and therefore cannot be proposed as non-invasive tests; rather, they provide molecular candidates for tissue-level characterization and for mechanistic follow-up. In contrast to single-marker approaches, they were selected using an unbiased multi-method framework incorporating random forest,SVM-RFE, and the MCC algorithm implemented in cytoHubba and validated in an independent glomerular cohort, albeit with moderate discrimination (AUC 0.72–0.80). Because FSGS is defined histologically, markers measured in the same biopsy specimen are not intended to replace histological assessment; their added value would lie in the molecular dimension that light microscopy alone cannot provide. We hypothesize that such markers could eventually help to separate FSGS from minimal change disease in diagnostically ambiguous biopsies and to stratify patients by the activity of cell-cycle and mitotic programs, and they nominate candidate therapeutic targets such as AURKA; these hypotheses are untested, as the present study design cannot evaluate diagnostic separation (no MCD-only cohort was analyzed) or patient stratification (no treatment-response or outcome data are available). Prospective studies linking these molecular readouts to treatment response are needed before clinical utility can be claimed.

This study presents certain limitations. Firstly, owing to restricted sequencing data availability, the sample sizes of both FSGS patients and controls were relatively small, potentially limiting the generalizability of findings to broader populations. Therefore, future research should encompass larger and geographically diverse cohorts to independently validate these outcomes. Second, obtaining healthy renal tissue as controls remains challenging, and the datasets used lacked demographic matching (e.g., age and sex). Future studies should ensure appropriate demographic matching. Third, the effect sizes of AURKA and CENPM were modest (about 1.3-fold), and diagnostic performance decreased in the independent validation cohort (AUC 0.72–0.80); larger, platform-matched cohorts are therefore required. Related to this, the supervised feature-selection steps were originally performed once on the full training cohort, and fold-wise re-analysis showed that AURKA was recovered in 36 of 50 cross-validation folds by both supervised methods whereas CENPM was recovered in only 4; the MDG > 1.0 threshold was not met within any single fold, because Gini importance scales with cohort size (S7 Table). Both genes — CENPM in particular — should therefore be regarded as exploratory. No threshold-based classification metrics are reported for the external cohort, because a cut-off calibrated on one profiling platform cannot be transferred to another; the previously reported external sensitivity and specificity values corresponded to the Youden-optimal point of the validation ROC itself and are retained only as a descriptive note. Fourth, this study was based on transcriptomic data alone; protein-level and functional validation of AURKA and CENPM was not performed and represents a key direction for future work. Public protein resources provide partial support: the Human Protein Atlas documents low-level protein expression of both genes in normal human kidney (tubular cells; glomerular staining not detected in healthy tissue), as expected for cell-cycle-associated genes in quiescent glomeruli (www.proteinatlas.org). Fifth, the training FSGS group included six cases with overlapping FSGS/MCD features, as annotated by the ERCB; a sensitivity analysis excluding these cases confirmed the robustness of the findings (S4 Table), although the inclusion of borderline diagnoses may have modestly diluted the observed effect sizes. Sixth, all analyses were restricted to kidney tissue: blood and urine samples from the same patients were not available, and the two genes were therefore not evaluated as non-invasive blood- or urine-based markers. Similarly, the public datasets provide limited clinical annotation, so we could not determine whether AURKA or CENPM expression differs between treatment-responsive and treatment-resistant patients, or whether these genes could contribute to patient stratification. These questions are clinically important and remain to be addressed in prospective cohorts with matched biospecimens and treatment outcome data. Seventh, the CIBERSORT estimates rely on the LM22 leukocyte reference matrix, which contains no intrinsic glomerular cell types; the inferred fractions should therefore be read as relative immune-enrichment signals rather than absolute cellular proportions. Marker-gene concordance checks (S8 Table) support the monocyte and activated NK cell signals but not the naive B cell or CD8 T cell estimates, and cell-type-resolved (single-cell or spatial) profiling is required for confirmation.

## Conclusions

In summary, by integrating WGCNA and machine learning methods, this study identified AURKA and CENPM as exploratory candidate tissue biomarkers — AURKA being the more robustly supported of the two — and demonstrated their associations with ICI. These findings are hypothesis-generating and require independent validation.

## Supporting information

S1 Dataset Raw series matrix file of the training cohort (GSE104948, GPL22945), as deposited in GEO. (GZ)

S2 Dataset Raw series matrix file of the validation cohort (GSE129973, GPL17586), as deposited in GEO. (GZ)

S1 Table Detailed sample information for the training cohort (GSE104948) and the independent validation cohort (GSE129973). (XLSX)

S2 Table Complete list of the 39 candidate genes from the green-yellow (greenyellow) module. (XLSX)

S3 Table Per-sample quality-control statistics (median, IQR, detected features, and PCA coordinates) for both cohorts. (XLSX)

S4 Table Sensitivity analysis of AURKA and CENPM expression in the training cohort after excluding the six overlapping FSGS/MCD cases. (XLSX)

S5 Table Per-sample CIBERSORT deconvolution results (22 immune cell fractions, P-value, Pearson correlation, and RMSE) for the 37 training samples. (XLSX)

S6 Table Robustness of random-forest feature selection to the random seed and the MDG threshold (eight seeds × three thresholds); the intersection of RF, SVM-RFE and cytoHubba MCC results was AURKA and CENPM in all 24 configurations. (XLSX)

S7 Table Fold-wise stability of supervised feature selection: random forest and linear SVM-RFE re-run independently inside the training portion of 50 outer folds (10 repeats of five-fold stratified cross-validation of the 37 training samples), with per-gene selection frequencies and per-fold detail. (XLSX)

S8 Table Immune-infiltration analyses with multiple-testing correction: between-group comparisons of the inferred cell fractions and gene–cell Spearman correlations under both sample sets (full training cohort, n = 37; significance-filtered subset, n  = 12), and marker-gene concordance of the CIBERSORT estimates. (XLSX)

S9 Dataset Analysis scripts and supporting files (README with software and package versions; preprocessing, quality control, threshold audit, fold-wise stability, supplementary table generation, marker-gene concordance, provenance checks and Fig 6B regeneration scripts; CIBERSORT input and output files). (ZIP)

S1 Fig Quality control of the training cohort (GSE104948). (A) Box plots of log2-transformed expression values for each sample, colored by group. (B) Principal component analysis computed on the top 8,000 most variable genes. (C) Hierarchically clustered heatmap of pairwise Pearson correlations between samples; the color band beneath the heatmap indicates group membership. Statistics: PCA was performed on the top 8,000 most variable genes (variance across the 37 analyzed samples); sample-to-sample correlations were calculated as Pearson coefficients on log2-transformed values.
(TIF)

S2 Fig Quality control of the independent validation cohort (GSE129973). (A) Box plots of log2-transformed expression values (transcript-cluster level) for each sample, colored by group. (B) Principal component analysis computed on the top 8,000 most variable transcript clusters. (C) Hierarchically clustered heatmap of pairwise Pearson correlations between samples; the color band beneath the heatmap indicates group membership. Statistics: PCA was performed on the top 8,000 most variable transcript clusters (variance across the 40 analyzed samples); sample-to-sample correlations were calculated as Pearson coefficients on log2-transformed values.
(TIF)
