# Supplementary material for: Integrated transcriptomic analysis and machine learning identify AURKA and CENPM as candidate biomarkers in focal segmental glomerulosclerosis
Source: PLoS One. 2026 Nov 10;21(11):e0361466. doi: 10.1371/journal.pone.0361466 (PMC13750537; doi:10.1371/journal.pone.0361466)
Supplement: Supplementary file 4 [file pone.0361466.s015.docx]

# Response to Reviewers

**Manuscript title:** Integrated transcriptomic analysis and machine learning identify AURKA and CENPM as candidate biomarkers in focal segmental glomerulosclerosis

**Journal:** PLOS ONE

**Academic Editor:** Prof. Claude Prigent

**Manuscript ID:** PONE-D-26-33048

**Authors:** Lin Xiaomei¹, Huang Guofang¹,², Sun Qing¹,², Zhang Xikui³*

¹Fujian University of Traditional Chinese Medicine, Fuzhou, Fujian, China

²Rehabilitation Hospital Affiliated to Fujian University of Traditional Chinese Medicine, Fuzhou, Fujian, China

³The Second Affiliated Hospital of Fujian University of Traditional Chinese Medicine, Fuzhou, Fujian, China

*Corresponding author: Zhang Xikui (zhangxk2026@outlook.com)

Dear Prof. Prigent,

We thank you and the two reviewers for the careful and constructive evaluation of our manuscript. The comments identified several substantial issues — in particular the independence of the validation cohort, the risk of overfitting in a small training set, and the framing of our findings — and addressing them has materially improved the work. We have revised the manuscript accordingly; all changes are highlighted in the marked-up copy, and a clean version is provided.

Three changes are central to this revision:

**1. The external validation cohort has been replaced, and the validation is now genuinely independent.** Following Reviewer 2's observation, we re-examined the provenance of the datasets. GSE104948 and GSE47183 are indeed derived from the same patient series (the European Renal cDNA Bank, ERCB): the GSE104948 record states that its samples were "included under previous GEO submissions — GSE47183 (+GSE32591)". On re-annotation, the two series share **seven patients** (FSGS915/917/918/927 and TN995/996/997), and 12 of the 27 samples (44%) of the GSE47183 "validation" set come from patients already present in the training set; GSE47183 additionally contains technical replicates of the same biopsy (its 14 "controls" represent only five patients). We therefore **discarded GSE47183 altogether** and repeated external validation in **GSE129973** (Nanjing University; 20 FSGS vs 20 controls; glomerular tissue), a cohort from a different institution and biobank with **no patient overlap** with the training set. As a consequence, the reported external performance is lower but honest: **AURKA AUC 0.797 (95% CI 0.661–0.934), CENPM AUC 0.720 (95% CI 0.553–0.887)**, versus 0.907/0.830 in the overlapping cohort.

**2. The study has been repositioned.** The title, abstract, results, discussion and conclusion now present AURKA and CENPM as **exploratory tissue-based candidate biomarkers** and hypothesis-generating findings, not as established diagnostic markers or non-invasive tests.

**3. The training cohort is now fully disclosed.** Six of the 16 training FSGS samples carry overlapping FSGS/MCD annotations in the original ERCB series; this is now stated in the Methods and in the Limitations, with a sensitivity analysis provided as S4 Table.

We are grateful for the opportunity to revise the manuscript and hope it is now suitable for publication in PLOS ONE.

# Part A. Editorial requirements

## A1. Formatting and file naming

*Editor: Please ensure that your manuscript complies with the PLOS ONE format requirements, including the file naming rules.*

**Response.** The manuscript has been checked against the PLOS ONE formatting templates, and file naming has been standardised: main figures as `Fig1.tif`–`Fig7.tif`, Supporting Information as `S1_Table.xlsx`–`S6_Table.xlsx` and `S1_Fig.tif`–`S2_Fig.tif`. Each figure now has its own caption **in the manuscript text**, placed directly after the paragraph in which the figure is first cited, as required by the PLOS ONE template; the captions are not embedded in the image files, in line with PLOS ONE policy.

**Changes in the manuscript:** Figure section rebuilt; all figure legends rewritten (one per figure, each ending with a brief *Statistics* sentence).

## A2 and A3. Funding information and financial disclosure

*Editor: We noticed that the funding information you provided in the "Funding Information" and "Financial Disclosure" sections does not match. Please ensure that you provide the correct grant number(s) for awards that you received for this study. Please also answer the following questions (a–d).*

**Response.** We apologise for the inconsistency in the original submission. **The study received no specific funding**, and the two entries have now been made consistent.

**(a) Sources of funding** (including grants and material support): the authors received no specific funding for this work. No funds or material support were received from any funding agency or organisation, including the authors' institution.

**(b) Role of the funder:** not applicable — there was no funding source, and therefore no funder had any role in study design, data collection and analysis, decision to publish, or preparation of the manuscript.

**(c) Payments to authors from any funder:** none. No author has received any salary, payment or other benefit from any funding body.

**(d) Explicit statement:** *The authors received no specific funding for this work.*

**Changes in the manuscript:** the Funding section now reads *"The authors received no specific funding for this work."* We kindly ask the editorial office to update the corresponding fields of the submission system accordingly.

## A4. Data availability

*Editor: In the online submission form you stated "[Further inquiries can be directed to the corresponding author]". All PLOS journals require that all data underlying the findings be made freely available.*

**Response.** We have replaced the data availability statement with an explicit pointer to the public repositories: the training cohort is available in GEO under **GSE104948** and the independent validation cohort under **GSE129973**. In addition, per-sample data underlying every summary statistic reported in the manuscript are provided as Supporting Information (per-sample quality-control statistics as S3 Table, per-sample CIBERSORT deconvolution results as S5 Table, and per-sample sample-level information as S1 Table).

**Changes in the manuscript:** Data availability statement rewritten (Section 10).

## A5. A caption for each figure

*Editor: Please add a caption for each figure in the manuscript.*

**Response.** We confirm that every main figure now has its own caption **in the manuscript**, with each caption placed directly after the paragraph in which the figure is first cited; the figure files themselves contain no caption text, in line with PLOS ONE policy. All legends were rewritten from scratch to match the actual panel structure of the figures, and each statistical legend ends with a short *Statistics* sentence describing the test used.

**Changes in the manuscript:** Fig 1–Fig 7 legends (each placed directly after its first citation in the text).

## A6. Titles of supporting information files and corresponding in-text citations

*Editor: Please add the titles of the Supporting Information files at the end of the manuscript and update the in-text citations accordingly.*

**Response.** The Supporting Information section now lists all eight files with full titles, in numerical order — Tables S1–S6 followed by Figures S1–S2 — and every item is now cited in the main text: S1 (sample information and cohort independence, Methods → Data Collection; ), S2 (the 39 candidate genes, Results), S3 and Figures S1–S2 (quality control, Methods → Data Collection), S4 (sensitivity analysis, Limitations), S5 (CIBERSORT per-sample results, Methods → Immune infiltration, and the Fig 6 legend), S6 (robustness of feature selection, Results → Feature selection).

**Changes in the manuscript:** Supporting Information section reorganised; in-text citations added in the Methods and Results.

## A7. The supplementary file that could not be opened

*Editor: We were unable to open your supplementary file [._GSE104948sample.xlsx]. Please modify and re-upload as appropriate.*

**Response.** Thank you for flagging this. The file we were unable to open, `._GSE104948sample.xlsx`, was not a data file: it is a macOS resource-fork artefact (*AppleDouble*) that was inadvertently included when the original supplementary file was uploaded — a known issue when files are packaged on macOS. The artefact has been removed. The data file itself (`GSE104948sample.xlsx`) contained only a sample list (group assignment and GEO accession for the 37 training samples); this information is now provided in a **substantially expanded form as S1 Table**, which lists the GEO accession identifier, the original ERCB annotation, the analysis group, the diagnosis/control type and the sensitivity-analysis status for **every sample of both cohorts** (37 training + 40 validation samples). All supplementary files have been verified to open correctly.

**Changes in the manuscript:** Supporting Information section (eight files, PLOS-compliant naming); the previous file is no longer part of the submission.

## A8 and A9. The two points highlighted in the editorial letter

*Editor: Reviewer 1 said "I find it hard to imagine that such a small difference could be used as a clinical diagnostic marker." Reviewer 2 pointed out that "the enrichment and immune deconvolution results support correlation but do not confirm that AURKA and CENPM regulate the proposed pathways or immune infiltration."*

**Response.** Both points are addressed in detail in our responses to **Reviewer 1, comment 2** and **Reviewer 2, comment 5**, respectively. In brief: (i) we now explicitly acknowledge the modest effect size and report the honest, lower discrimination obtained in the independent cohort, and we reposition the genes as exploratory tissue-based candidates whose value lies in the convergence of independent analytical lines rather than in single-gene effect size; (ii) all enrichment and immune-deconvolution results are now described as association-only evidence, with dedicated statements in the Methods, the Results and the Discussion that these analyses "do not establish a regulatory relationship".

# Part B. Reviewer 1

## R1-1. Training and external evaluation in separate datasets

*Reviewer 1 comment 1: "I appreciate that the authors first trained their model on an initial dataset and then evaluated its efficiency/efficacy in a different dataset."*

**Response.** We thank the reviewer for this comment. In response to Reviewer 2 (comment 1), we have taken this principle further: the external evaluation is now performed in a cohort that is not only a different dataset but, unlike the original GSE47183 series, also **independent at the patient level** (different institution and biobank, no overlapping patients; see Part C, comment 1).

**Changes in the manuscript:** External validation section; Tables and Fig 7 updated to GSE129973.

## R1-2. The magnitude of the expression difference and the mRNA-to-protein gap

*Reviewer 1 comment 2: "The gene expression differences revealed by the authors' analysis are very small (about 5% to 7% of controls). Even if statistically significant, I find it hard to imagine that such a small difference could be used as a clinical diagnostic marker. Moreover, these small differences relate to mRNA levels and may have no effect on the corresponding protein levels, let alone on cellular function. These two points should at least be discussed."*

**Response.** We fully agree that both points deserve explicit discussion, and we have addressed them in the revised manuscript.

**(i) Effect size and diagnostic performance.** We now state the effect sizes explicitly (training cohort: log2FC 0.47 for AURKA and 0.40 for CENPM, i.e. approximately 1.3-fold; independent cohort: 0.18 and 0.07) and report the corresponding discrimination honestly. Crucially, when the overlapping validation set was replaced by the truly independent cohort, the external performance dropped from AUC 0.907/0.830 to **0.797 (95% CI 0.661–0.934) / 0.720 (95% CI 0.553–0.887)** — at the Youden-optimal cut-off this corresponds to 90.0% sensitivity with only **60.0% specificity** for AURKA and **55.0%** for CENPM (accuracy 75.0% and 72.5%), which is clearly insufficient for a diagnostic test. We have therefore **removed any claim that these genes constitute a diagnostic biomarker** and instead present them as exploratory tissue-based candidates. We consider it more valuable to report the lower, honest performance in a genuinely independent cohort than to retain inflated estimates contaminated by sample overlap. To make this contrast explicit and to avoid any impression that the training-cohort figures stand alone, the Results now describe the training-cohort finding as "high discriminative performance in the training cohort, in contrast to the lower performance in the independent validation cohort".

**(ii) mRNA versus protein.** We have added a dedicated limitation stating that the study is based on transcriptomic data alone and that protein-level and functional validation was not performed, and we have added an explicit note on the interpretation of mRNA-level findings: a 1.3-fold transcript change need not translate into a proportional change in protein abundance or activity. To provide at least indirect protein-level context, we consulted the **Human Protein Atlas**: both AURKA (antibodies HPA002636/CAB001454) and CENPM (antibody HPA042404) have antibody-based evidence at the protein level in human kidney, with low staining in tubular cells and consensus RNA expression of 2.3 nTPM and 2.1 NX, respectively; notably, glomerular staining was not detected in healthy tissue, which is the expected baseline for cell-cycle-associated genes in quiescent glomeruli. We are explicit that this is contextual support and **not** a substitute for disease-state, glomerulus-resolved protein validation, which remains future work.

**Changes in the manuscript:** Results (ROC section) — training-cohort wording revised ("excellent diagnostic performance" → "high discriminative performance in the training cohort, in contrast to the lower performance in the independent validation cohort"); Results (external validation) — effect sizes, AUCs with confidence intervals and classification performance added; Limitations, third and fourth points — modest effect size, mRNA-to-protein caveat and HPA evidence.

## R1-3. The description of AURKA expression

*Reviewer 1 comment 3: "In the discussion, the authors state that Aurora A is 'highly expressed in the FSGS cohort'. According to the data provided, this is not the case."*

**Response.** The reviewer is correct, and we apologise for this overstatement. The sentence now reads that AURKA was **"moderately upregulated"** in the FSGS cohort, which matches the observed effect size. We have also searched the entire manuscript for comparable expressions and replaced them with wording consistent with the data.

**Changes in the manuscript:** Discussion (AURKA paragraph): "highly expressed" → "moderately upregulated".

## R1-4. How tissue-derived markers relate to histopathology, and whether blood was analysed

*Reviewer 1 comment 4: "Since the authors use FSGS biopsy samples, I wonder how the identification of these two markers could improve histopathological examination. Have the authors analysed blood samples from the patients?"*

**Response.** Two separate questions are involved.

**(i) Blood samples.** No — no blood (or urine) samples were available for the patients in the public datasets used here. Both genes are therefore explicitly **not** proposed as non-invasive blood- or urine-based markers. This is now stated as a limitation.

**(ii) How such markers could add value on top of histopathology.** We have added a dedicated passage to the Discussion clarifying the intended positioning. Because FSGS is defined histologically, markers measured in the same biopsy specimen are not intended to replace histological assessment. Their added value lies in the molecular dimension that light microscopy alone cannot provide: helping to separate FSGS from minimal change disease in diagnostically ambiguous biopsies, stratifying patients by the activity of cell-cycle and mitotic programmes, and nominating candidate therapeutic targets such as AURKA. We also note explicitly that prospective studies linking such molecular readouts to treatment response are required before clinical utility can be claimed.

**Changes in the manuscript:** Limitations, sixth point (blood/urine samples not available); Discussion (comparison with previously reported biomarkers) — new passage on positioning relative to histopathology.

## R1-5. Patient sample status and patient stratification

*Reviewer 1 comment 5: "The authors correctly mention treatment-refractory patients. What is the status of the patient samples used in this study? Could Aurora A and/or CENP-M be used for patient stratification?"*

**Response.** The public datasets used here (GSE104948 and GSE129973) provide only the diagnosis (FSGS versus control) with limited clinical annotation; treatment response, disease stage and follow-up outcome are not available for most samples. We therefore **cannot** determine whether AURKA or CENPM expression differs between treatment-responsive and treatment-resistant patients, or whether they could contribute to patient stratification. Rather than speculate, we state this as an explicit limitation and point out that answering the question requires prospective cohorts with matched biospecimens and treatment-outcome data.

**Changes in the manuscript:** Limitations, sixth point.

## R1-6 (overall assessment). Significance for the readership

*Reviewer 1 (concluding remark): "Aurora A and CENP-M may indeed be promising FSGS biomarkers, but the differences observed between patient cohorts and healthy individuals are very small, which in my view makes this possibility unlikely, and this also raises questions about the significance of the paper for PLOS readers."*

**Response.** We have taken this concern seriously and have restructured the framing of the paper accordingly. The study is now presented explicitly as **hypothesis-generating** work whose contribution is not a diagnostic test but: (i) the convergent identification of two cell-cycle genes by three independent analytical routes (WGCNA module membership, random forest, SVM-RFE, and PPI-based MCC) — the intersection being robust across all 24 seed × threshold configurations tested; (ii) an independent-cohort expression profile with an honest, moderate discriminative performance; and (iii) a mechanistic link to cell-cycle and mitotic programmes in a disease whose podocyte loss is known to involve mitotic catastrophe. We hope this repositioning, together with the explicit limitations, makes the value of the work clear to the readership — as a starting point for protein-level and functional follow-up rather than as a clinical claim.

**Changes in the manuscript:** Title; Abstract (Conclusion); Results (final paragraph); Discussion; Conclusion — consistent repositioning as exploratory tissue-based candidate biomarkers.

# Part C. Reviewer 2

## R2-1. Sample overlap between the training and validation cohorts

*Reviewer 2 comment 1: "The GEO record for GSE104948 clearly states that the samples were previously included in GSE47183, which is used here for external validation. Please provide the exact sample identifiers, verify the independence of patients and samples, and if there is overlap, repeat the validation using a truly independent cohort."*

**Response.** The reviewer is entirely correct, and the problem proved to be more extensive than the GEO record alone suggests. Our verification found that:

- The GSE104948 series matrix states explicitly that its samples were "included under previous GEO submissions — GSE47183 (+GSE32591)"; both are ERCB collections, and GSE104948 represents a re-annotation of the same biological material (GSM 2810xxx vs GSM 1146xxx).

- At the patient level, the training and "validation" subsets share **seven patients** (FSGS915, FSGS917, FSGS918, FSGS927, TN995, TN996, TN997). Twelve of the 27 GSE47183 samples (44%) therefore come from patients already used for training.

- GSE47183 additionally contains technical replicates of the same biopsy: its 14 samples labelled as controls correspond to only five patients (TN2 ×3, TN4 ×3, TN995 ×2, TN996 ×4, TN997 ×4). Of its nine "new" FSGS samples, eight also belong to the GSE104948 series.

Accordingly, **we have discarded GSE47183 in its entirety** and re-performed external validation in **GSE129973** (Nanjing University Second Affiliated Hospital; 20 FSGS vs 20 controls; glomerular tissue), a cohort from a different institution and biobank that shares no patients with GSE104948.

|  | **Original validation set (GSE47183)** | **New validation set (GSE129973)** |
| --- | --- | --- |
| Source | ERCB (same biobank as training set) | Nanjing University (independent) |
| Patient overlap with training set | 7 shared patients; internal technical replicates | None |
| Samples | 13 FSGS + 14 controls (with replicates) | 20 FSGS + 20 controls |
| AURKA | AUC 0.907; adj. P = 0.060 | AUC 0.797; adj. P = 0.012 |
| CENPM | AUC 0.830; adj. P = 0.204 | AUC 0.720; adj. P = 0.22 |

Exact sample identifiers, together with the original ERCB annotation and the analysis group for every sample, are provided in **S1 Table** (S1a: 37 training samples; S1b: 40 validation samples).

We chose to report the honest, lower performance in a truly independent cohort rather than retain inflated estimates contaminated by sample overlap.

**Changes in the manuscript:** Methods (Data Collection) — cohort description, statement that no patient overlap exists, and citation of S1 Table; Results (external validation) — all values re-computed; Fig 7 replaced with the GSE129973 analysis; Abstract, Discussion and Limitations updated throughout.

## R2-2. Risk of overfitting and the reporting of performance

*Reviewer 2 comment 2: "The small sample size also makes the feature-selection process and the reported performance prone to overfitting. Please clarify whether all preprocessing, gene filtering, WGCNA, random forest and SVM-RFE selection were performed in the training data, and explain the discrepancy between the 8,000 and 12,074 genes in the Methods and Results, as well as the discrepancy between the random-forest importance threshold and Fig 3. Exact external AUC values, confidence intervals and classification performance should be reported rather than just described as 'above 0.8'."*

**Response.** We address the four points in turn.

**(a) Independence of all modelling steps.** We have made this explicit: **all preprocessing, gene filtering, WGCNA network construction and feature selection (random forest, SVM-RFE and cytoHubba MCC) were performed exclusively on the training cohort**; the independent validation cohort was processed separately and used only for evaluation. For completeness, the preprocessing and quality-control steps were as follows: **quality control** of both cohorts comprised inspection of per-sample expression distributions, principal component analysis and hierarchical clustering, and no sample was excluded (Figures S1–S2; per-sample statistics in S3 Table); **preprocessing** used the depositor-provided, log2-scale gene-level matrix (Human Entrez Gene ID custom CDF version 19; 12,074 genes), with Entrez identifiers annotated to gene symbols and duplicates collapsed (12,045 genes); gene filtering/selection (the top 8,000 most variable genes) and all downstream modelling were then performed exclusively within the training cohort, while the validation cohort (GSE129973, HTA 2.0) was processed separately. All of these steps are now described explicitly in the Methods (Data Collection and Preprocessing; WGCNA).

**(b) 8,000 versus 12,074 genes.** These numbers refer to two different steps and are not in conflict: 12,074 genes were retained after preprocessing of GSE104948, and the **top 8,000 most variable genes** (of these 12,074) were used as input for WGCNA network construction. The wording is now identical in the Methods and the Results ("the top 8,000 most variable genes (out of 12,074 preprocessed genes)").

**(c) Random-forest threshold and Fig 3.** The random-forest model used 500 trees and genes with a Mean Decrease Gini (MDG) > 1.0 were retained, yielding five candidate genes; this is now stated both in the Methods and in the Results, and Fig 3A is accompanied by a legend describing exactly what is plotted and what the threshold represents. To address the overfitting concern directly, we performed a systematic robustness analysis: across **8 random seeds × 3 MDG thresholds (1.0, 0.9, 0.8) = 24 configurations**, the intersection of the three methods (random forest, SVM-RFE, cytoHubba MCC) was **AURKA and CENPM in every single configuration**. This is now reported in the Results and provided in full as S6 Table.

**(d) Exact external performance.** We now report the exact values with confidence intervals and classification performance for the independent cohort: **AURKA AUC 0.797 (95% CI 0.661–0.934)**, and **CENPM AUC 0.720 (95% CI 0.553–0.887)**. At the Youden-optimal cut-off, AURKA achieved 90.0% sensitivity and 60.0% specificity (overall accuracy 75.0%), and CENPM 90.0% sensitivity and 55.0% specificity (accuracy 72.5%). For completeness, the corresponding training-cohort values are AURKA AUC 0.949 (0.883–1.000) and CENPM AUC 0.943 (0.875–1.000). We deliberately do not describe performance as "above 0.8" anywhere.

**Changes in the manuscript:** Methods (Data Collection and Preprocessing — quality control and preprocessing now explicit; Screening of Core Genes; Statistical analysis; Immune infiltration) — statements of training-only procedures, of the preprocessing steps and of the Youden-based metrics; Results (WGCNA; Feature selection; External validation) — unified gene counts, five-gene RF threshold, 24-configuration robustness result, exact AUCs with CIs and classification performance; Fig 3 and Fig 7 legends; S6 Table.

## R2-3. Positioning as exploratory tissue biomarkers

*Reviewer 2 comment 3: "Because the study analyses kidney tissue expression rather than blood or urine, the findings should be defined as exploratory tissue biomarkers rather than a non-invasive diagnostic test or established evidence for clinical application."*

**Response.** We agree and have repositioned the study throughout. The phrase "exploratory tissue-based candidate biomarkers" now appears in the Abstract (Conclusion), the Results and the Discussion; all statements implying established clinical utility or non-invasive testing have been removed. As noted above, we also report explicitly that the genes cannot serve as blood- or urine-based markers with the data available.

**Changes in the manuscript:** Title (see R2-4); Abstract; Results (final paragraph of the validation section); Discussion; Conclusion.

## R2-4. The title and the "multi-omics" claim

*Reviewer 2 comment 4: "The title promises multi-omics integration, but the analyses presented appear to rely on transcriptomic data and downstream computational analysis."*

**Response.** The reviewer is right: our data are transcriptomic, and downstream enrichment, network and deconvolution analyses do not constitute additional omics layers. The title has been changed accordingly.

**Changes in the manuscript:** Title — now "Integrated **transcriptomic analysis** and machine learning identify AURKA and CENPM as **candidate biomarkers** in focal segmental glomerulosclerosis" (sentence case per the PLOS ONE title-page template; previously "Multi-Omics … Core Biomarkers" in title case).

## R2-5. Enrichment and deconvolution results support association, not regulation

*Reviewer 2 comment 5: "The enrichment and immune deconvolution results support association but do not confirm that AURKA and CENPM regulate the proposed pathways or immune infiltration. Please discuss possible cell-composition effects, the applicability of LM22 to glomerular tissue, and the need for independent experimental validation."*

**Response.** We agree entirely, and we have addressed this at three levels.

**(i) Wording.** Statements about enrichment and immune infiltration have been changed from regulatory language (for example, "further expand the regulatory landscape of APC/C-associated pathways") to association language ("further support the involvement of … in APC/C-associated pathways"), and the Results now state explicitly that the observed associations "do not imply causality".

**(ii) Methodological statements.** The Methods now state that these analyses "quantify association only and do not establish a regulatory relationship", and the correlation analyses underlying Fig 6B are described in full (cell–cell associations by Pearson correlation; gene–cell associations by Spearman rank correlation, with block size/link width encoding association strength and link colour encoding the significance class).

**(iii) Two specific caveats requested by the reviewer.**

- *Cell-composition effects.* CIBERSORT estimates relative cell fractions from bulk tissue expression, so differences between groups may partly reflect a shift in tissue cell composition rather than a change in per-cell expression of AURKA/CENPM; because both genes are expressed at low levels in quiescent glomeruli, a small number of infiltrating cells could disproportionately influence their apparent correlation with immune cell fractions.

- *Applicability of LM22 to kidney.* The LM22 signature matrix was derived mainly from peripheral blood leukocytes and therefore does not fully represent the immune cell populations of the kidney; glomerular tissue contains relatively few resident leukocytes, and several cell types prominent in blood are inferred from a limited number of marker genes. Relative differences remain internally comparable because all samples were processed with an identical pipeline and the same signature matrix. We also note that CD4+ naïve T cells and resting eosinophils were not detected in these samples.

- *Need for independent experimental validation.* We now state explicitly that establishing that AURKA and CENPM actually regulate the pathways or immune profiles with which they correlate will require independent experimental work, including detection of the two proteins in situ in FSGS biopsies, cell-type-resolved profiling, and functional experiments in podocytes or glomerular endothelial cells.

**Changes in the manuscript:** Methods (Statistical analysis; Immune infiltration); Results (immune infiltration paragraph); Discussion (immune paragraph; two caveats paragraph; comparison with previously reported biomarkers).

## R2-6. Comparison with previous biomarkers and a complete, reproducible workflow

*Reviewer 2 comment 6: "The manuscript would be substantially strengthened by a clearer comparison with previous FSGS biomarker studies and by a complete, reproducible description of the analytical workflow."*

**Response.** We have added both.

**(i) Comparison with previously reported biomarkers.** A dedicated passage now compares our findings with circulating and urinary candidates — soluble urokinase-type plasminogen activator receptor (suPAR), anti-nephrin autoantibodies, and urinary NGAL and KIM-1 — noting that those markers have the advantage of being measurable non-invasively but that their specificity for FSGS, particularly in distinguishing FSGS from other proteinuric glomerulopathies, remains debated. We state that AURKA and CENPM were derived from glomerular tissue and therefore cannot be proposed as non-invasive tests, and that they provide molecular candidates for tissue-level characterisation and mechanistic follow-up. We also highlight that, unlike single-marker approaches, they were selected within an unbiased multi-method framework and validated in an independent glomerular cohort.

**(ii) Reproducible workflow.** The Methods now specify the key parameters of every step (soft-thresholding power 8 with R² = 0.85; minimum module size 30; module membership and gene significance > 0.5; 500 decision trees with MDG > 1.0; five-fold cross-validation for SVM-RFE; top-20 MCC genes; 1,000 permutations and quantile normalisation for CIBERSORT), together with the R packages used (WGCNA in TBtools II; e1071; randomForest; clusterProfiler; ReactomePA; pROC; ggplot2; R version 4.2.1). The Methods also describe the preprocessing and quality-control stage with the same specificity: per-sample expression-distribution inspection, PCA and hierarchical clustering (no sample excluded; Figures S1–S2 and Table S3); the depositor-provided log2-scale gene-level matrix (Entrez CDF version 19; 12,074 genes), with Entrez identifiers annotated to gene symbols and duplicates collapsed (12,045 genes); and variance-based selection of the top 8,000 most variable genes. In addition, the Data availability statement now notes that **all analysis scripts (preprocessing, quality control, WGCNA, feature selection, ROC analysis and immune-infiltration analysis) are available from the corresponding author upon reasonable request**.

**Changes in the manuscript:** Discussion (comparison with previously reported biomarkers — new passage); Methods throughout (including Data Collection and Preprocessing: quality control and gene filtering); Data availability statement.

# Part D. Additional corrections made during revision

While preparing this revision we identified and corrected several further issues; we report them here for full transparency.

**D1. Percentage of significant CIBERSORT deconvolutions.** The Methods previously left the number of retained samples unspecific. CIBERSORT was run on all 37 training samples (1,000 permutations, quantile normalisation); significant deconvolution (P < 0.05) was obtained in **12 of 37 samples (8 FSGS and 4 controls)**. Per-sample values are provided in S5 Table.

**D2. Terminology.** "GSEA" and "ssGSEA" were used interchangeably in the original text; the analysis actually performed was GSEA (clusterProfiler, gene sets from the curated pathway (CP) subcollection of the MSigDB C2 collection, genes ranked by log2 fold change). The terminology has been unified, and the description of the gene-set source corrected.

**D3. Statistical label in Fig 6B.** During revision we re-computed the statistics underlying Fig 6B from the original per-sample data and found that the links between the hub genes and immune cell fractions were generated with **Spearman rank correlation** (the reported r and P values match Spearman exactly for all 40 links), whereas the embedded legend labelled them as "Mantel". The embedded legend has been corrected to "Spearman's p/r", and the corresponding Methods, Results and figure legend text now describe the two types of association correctly (cell–cell: Pearson; gene–cell: Spearman). The heat-map colour scale ("Pearson's r") was verified as correct and left unchanged.

**D4. Training-cohort composition.** Six of the 16 training FSGS samples carry overlapping FSGS/MCD annotations in the original ERCB series (GSM2810777–GSM2810782). This is now disclosed in the Methods and in the Limitations. A sensitivity analysis (S4 Table) shows that after excluding these six samples, AURKA remained strongly upregulated (log2FC 0.513, AUC 0.986) and CENPM was essentially unchanged (log2FC 0.356, AUC 0.933); the inclusion of these borderline cases therefore introduced a conservative bias rather than inflating the signal. The remaining samples of the series (diabetic nephropathy, pure MCD, and RPGN) were not included in any analysis.

**D5. S6 Table added.** A new supporting table reports the robustness of the three-method intersection across 8 random seeds × 3 MDG thresholds.

**D6. How the two panels of Fig 4 are defined.** The manuscript previously described Fig 4A and 4B as gene sets "associated with AURKA" and "associated with CENPM", which implied that a separate GSEA had been run for each gene. This was not the case: a single GSEA was performed on genes ranked by the FSGS-versus-control log2 fold change. We have therefore re-written the Methods, Results, Discussion and the Fig 4 legend to state this explicitly, and the two panels are now described as two functional groups of enriched pathways, with the link to the hub genes specified precisely: AURKA is a leading-edge (core-enriched) gene of the two APC/C gene sets shown in panel A, and CENPM is a leading-edge gene of all four gene sets shown in panel B. We have also relabelled the two panels of Fig 4 to describe the functional groups (‘Translation / APC/C programmes’ and ‘M phase / checkpoint programmes’) rather than a single gene, so that the figure no longer implies a per-gene analysis. No data were re-analysed and the enrichment plots themselves are unchanged.

We thank the editor and both reviewers again for their time and for comments that have substantially improved the rigour and the clarity of the manuscript. We would be glad to provide any further information or analyses that may be required.

Yours sincerely,

**Zhang Xikui**

The Second Affiliated Hospital of Fujian University of Traditional Chinese Medicine, Fuzhou, Fujian, China

zhangxk2026@outlook.com

On behalf of all authors (Lin Xiaomei, Huang Guofang, Sun Qing, Zhang Xikui)
