# Supplementary material for: Integrated transcriptomic analysis and machine learning identify AURKA and CENPM as candidate biomarkers in focal segmental glomerulosclerosis
Source: PLoS One. 2026 Nov 10;21(11):e0361466. doi: 10.1371/journal.pone.0361466 (PMC13750537; doi:10.1371/journal.pone.0361466)
Supplement: Supplementary file 5 [file pone.0361466.s016.docx]

# Response to Reviewers (Round 2)

**Manuscript ID:** PONE-D-26-33048R1

**Title:** Integrated transcriptomic analysis and machine learning identify AURKA and CENPM as candidate biomarkers in focal segmental glomerulosclerosis

**Journal:** PLOS ONE

**Academic Editor:** Prof. Claude Prigent

**Authors:** Lin Xiaomei, Huang Guofang, Sun Qing, Zhang Xikui*

*Corresponding author: Zhang Xikui (zhangxk2026@outlook.com)

Dear Prof. Prigent,

We thank you and Reviewer 2 for the continued careful scrutiny of our manuscript. The second-round comments again identified concrete points — in particular the provenance of the reported classification thresholds, the specification of the immune analyses, and the availability of the analysis code — and we have addressed each of them with new analyses and revised text rather than with reassurances alone. All changes are highlighted in the marked-up copy; a clean version is also provided.

The substantive changes in this revision are:

1. **The external validation is now reported threshold-free.** We re-derived the previously reported external sensitivity/specificity/accuracy values from the per-sample data and confirmed that they were computed at the Youden-optimal cut-off of the validation ROC itself, i.e., the threshold was selected on the data being evaluated. We acknowledge this as post-hoc threshold optimisation. External performance is now reported as AUC with 95% CI (threshold-free) as the primary measure; the previous classification metrics are no longer presented as validation results, and the manuscript now explains why an absolute cut-off cannot be transferred between the two profiling platforms.
2. **Feature-selection stability is now quantified within cross-validation folds.** The two supervised selection methods (random forest, SVM-RFE) were re-run independently inside the training portion of 50 outer folds (10 repeats of five-fold stratified CV of the 37 training samples). Selection frequencies per gene are reported in a new supporting table (S7 Table), and the claims attached to each gene have been tempered accordingly.
3. **The immune analyses are fully specified and FDR-corrected.** The exact sample set of every comparison and correlation is now stated (and flagged per sample in S5 Table), Benjamini-Hochberg correction is applied across the 20 detectable cell types and the 40 gene-cell tests, and a full-cohort analysis (n = 37; the basis of Fig 6A) is reported as the primary analysis, with the significance-filtered subset (n = 12) provided as an underpowered descriptive subset. All results, including those that do not survive correction, are reported (new S8 Table).
4. **All analysis scripts and software details are made directly available** (provided as Supporting Information, see R2-9), rather than on request.

## Part A. To the Academic Editor

This revision responds to the second-round comments of Reviewer 2; the responses follow in Part B. We confirm that: (i) no reference suggested by the reviewer has been added or removed in this round, and the reference list is unchanged; (ii) no retracted article is cited; (iii) the three required files are uploaded — the response letter (this document), the manuscript with tracked changes, and the clean revised manuscript. Because the study is computational, no laboratory protocol is applicable and no protocol has been deposited to protocols.io; instead, the complete analysis code, all per-sample data underlying the reported statistics and the full software version list are provided as Supporting Information (S9 Dataset), as described in R2-9.

## Part B. Response to Reviewer 2

### R2-1. Sample counts: the total number of control replicates is 16, not 14

**Reviewer comment:** "Please check the sample numbers, as the total number of control replicates listed is 16 rather than 14."

**Response:** The reviewer is correct, and the error was ours. In our previous response letter (Part C, R2-1) we described the GSE47183 control series as "14 samples labelled as controls", although the breakdown given in the same sentence — TN2 x3, TN4 x3, TN995 x2, TN996 x4, TN997 x4 — sums to 16. On re-checking the GSE47183 sample table, the controls comprise 16 samples from five patients (TN2 x3, TN4 x3, TN995 x2, TN996 x4, TN997 x4); the total was mis-stated as 14 in that sentence of the previous response letter. The error was confined to that sentence of the correspondence: GSE47183 was discarded in the previous revision, and no manuscript text, table or figure refers to it. The cohort descriptions that the manuscript actually relies on were re-verified sample by sample against S1 Table and are correct: training cohort GSE104948, 37 samples (16 FSGS vs 21 controls); validation cohort GSE129973, 40 samples (20 FSGS vs 20 controls).

**Changes:** The previous response is corrected here. No manuscript change was required (verified).

### R2-2. Gene numbers before and after removal of duplicates (12,074 vs 12,045)

**Reviewer comment:** "Please explain the change in gene numbers before and after removing duplicate genes (12,074 vs 12,045)."

**Response:** The two numbers refer to consecutive preprocessing steps, which the revised Methods now state explicitly. The depositor-provided log2-scale gene-level matrix (Human Entrez Gene ID custom CDF, version 19) contains 12,074 Entrez-level identifiers. During annotation to official gene symbols, 29 identifiers were dropped because they lacked a one-to-one current official gene symbol under the CDF v19 annotation (retired or merged Entrez IDs); the remaining 12,045 entries are unique gene-level identifiers. We additionally note that such re-annotation is reference-version dependent: mapping the same 12,074 identifiers against the current org.Hs.eg.db release (v3.17) returns 32 identifiers without a current symbol (12,042 unique symbols) — the same accounting to within a few identifiers. Consistently, the gene-level mixture matrix deposited for the CIBERSORT run contains 12,042 gene-level entries. The revised Methods sentence now reads: "The depositor-provided, log2-scale gene-level expression matrix (annotated using the Human Entrez Gene ID custom CDF, version 19) comprising 12,074 Entrez-level identifiers was used as input; during annotation to official gene symbols, 29 identifiers lacking a one-to-one current official gene symbol (retired or merged Entrez IDs) were removed, leaving 12,045 unique gene-level entries, from which the top 8,000 most variable genes were selected."

**Changes:** Methods (Data Collection and Preprocessing) — one sentence added. No analytical change.

### R2-3. Seed and threshold analyses are useful but do not resolve overfitting

**Reviewer comment:** "The seed and threshold analyses are useful, but do not resolve the overfitting problem in such a small cohort."

**Response:** We agree. The 24-configuration analysis (S6 Table; 8 seeds x 3 MDG thresholds) shows that the full-cohort selection pipeline returns AURKA and CENPM under every configuration, but it cannot indicate how often each gene would be selected from smaller subsets of the same data — which is the overfitting-relevant question. We have therefore added a fold-wise stability analysis: random forest (500 trees) and linear SVM-RFE were each re-run independently inside the training portion of 50 outer folds (10 repeats of five-fold stratified cross-validation of the 37 training samples), and per-gene selection frequencies were recorded for all 39 candidate genes (FAM64A mapped through its current official symbol PIMREG) (new S7 Table). The outcome is reported candidly. AURKA was among the top five genes in 38/50 folds (random forest) and 47/50 folds (SVM-RFE) and was selected by both methods in 36/50 folds (72%). CENPM was less stable: 33/50 (random forest), 4/50 (SVM-RFE), and 4/50 for both methods combined. We also verified that the MDG > 1.0 threshold is not fold-portable — Gini importance magnitudes scale with cohort size, and the threshold was met in 0/50 training folds — so the fold-wise analysis used a fixed top-5 rule mirroring the original five-gene random-forest subset. The cytoHubba-MCC step was not repeated fold-wise because it depends only on the (fixed) PPI topology of the candidate list, not on the samples. The revised text now states explicitly that AURKA is the more robustly selected of the two candidates, that CENPM's selection is partly configuration-dependent at this sample size, and that this — together with the modest external performance — is the quantitative basis for treating both genes, CENPM in particular, as exploratory candidates requiring independent confirmation. The Limitations section has been expanded accordingly.

**Changes:** Results (Feature selection) — new paragraph; new S7 Table (per-fold selection results); Limitations — expanded; Discussion — claims tempered.

### R2-4. Was feature selection repeated within each cross-validation fold, and was the reported classification threshold selected on training or validation data?

**Reviewer comment:** "Please explain whether feature selection was repeated in each cross-validation fold, and whether the reported classification threshold was selected using training or validation data."

**Response:** We answer both questions directly and describe the corrections made.

**(i) Feature selection.** In the workflow originally reported, feature selection was performed once on the full training cohort; the five-fold cross-validation associated with SVM-RFE was used only to choose the subset size, not to re-select features within folds. This was not stated explicitly in the original text — an omission we have corrected. The fold-wise analysis described in R2-3 now quantifies within-fold selection directly (AURKA: both methods 36/50 folds; CENPM: 4/50 folds).

**(ii) Classification threshold.** The reviewer's concern is well founded. We re-derived the reported external metrics from the per-sample data and confirmed that they correspond to Youden-optimal operating points of the validation ROC itself: for AURKA, 90.0% sensitivity / 60.0% specificity / 75.0% accuracy is exactly the Youden-optimal point of the validation ROC; for CENPM, two thresholds tie at the same Youden index and the reported 90.0% / 55.0% / 72.5% corresponds to the pROC tie-breaking rule. In other words, the threshold was selected on the data being evaluated — post-hoc threshold optimisation that we should have declared, and now correct as follows. (a) The external validation is reported threshold-free: AUC with 95% CI is the primary measure (AURKA 0.797, 95% CI 0.661-0.934; CENPM 0.720, 95% CI 0.553-0.887), and these values are unchanged because they involve no threshold. (b) The external sensitivity/specificity/accuracy values are no longer presented as validation results; they are retained only as a clearly labelled descriptive note stating that they describe the validation ROC's own optimal operating point and carry no independent-threshold interpretation. (c) We now state why no training-derived cut-off can be substituted: the two cohorts were profiled on different platforms (GPL22945 vs HTA 2.0) with non-overlapping log2 intensity scales. Applying the training-cohort Youden cut-off unchanged produces degenerate classification in the validation cohort (sensitivity 0/20 for both genes), and a z-score-standardised transfer likewise fails (AURKA sensitivity 10%); a platform-matched calibration cohort would be required for any deployable threshold. This point is now made in the Methods and the Limitations.

**Changes:** Methods (Statistical analysis); Results (External validation) rewritten; Limitations — threshold-transferability sentence added.

### R2-5. CIBERSORT: exactly which samples entered each analysis, and how multiple testing was handled

**Reviewer comment:** "Given that only 12 of 37 samples passed the CIBERSORT significance criterion, please specify exactly which samples were included in each comparison and correlation analysis, and explain how multiple testing was handled."

**Response:** All per-sample deconvolution results (22 cell fractions, CIBERSORT P value, deconvolution Pearson correlation, RMSE) are already provided per sample in S5 Table; the revision now flags, for every downstream analysis, exactly which samples were used. The 12 samples with significant deconvolution (P < 0.05, 1,000 permutations) are 8 FSGS samples (GSM2810777, GSM2810779, GSM2810780, GSM2810784, GSM2810787, GSM2810789, GSM2810790, GSM2810791) and 4 control samples (GSM2810798, GSM2810801, GSM2810806, GSM2810839). The revised Methods state the n of every analysis explicitly: group comparisons of cell fractions and gene-cell correlations use the 12 significance-filtered samples (8 FSGS vs 4 controls); a pre-specified sensitivity analysis repeats all comparisons on all 37 samples, with the per-sample significance flag carried in S5 Table. Multiple testing is now handled explicitly with Benjamini-Hochberg FDR correction — across the 20 detectable cell types (two types are zero in all samples) for group comparisons and across the 40 gene-cell Spearman tests — and every raw and adjusted P value is provided in a new S8 Table, under both sample sets. Fig 6A displays the full training cohort (n = 37). In addition, Fig 6B has been regenerated directly from the deposited per-sample deconvolution results (S5 Table) with the methods stated in the revised text, so that the figure, the Results text and S8 Table now share a single, verifiable data source.

We report the outcome candidly. Within the 12-sample subset, no group comparison and no gene-cell correlation survives FDR correction: the smallest adjusted P value is 0.051 (monocytes, activated NK cells, neutrophils), which is unsurprising at n = 12 (8 vs 4). In the full training cohort (n = 37), monocytes (BH-adjusted P = 7.9 x 10-5), naive B cells (0.0025), activated NK cells (0.0073), neutrophils (0.0084) and resting and activated dendritic cells (0.027) remain significantly different between FSGS and controls, and three of the 40 gene-cell tests survive FDR: CENPM-monocytes (rho = 0.61, adjusted P = 0.002), AURKA-monocytes (rho = 0.58, adjusted P = 0.003) and AURKA-activated NK cells (rho = 0.50, adjusted P = 0.022). The Results and Discussion now rest the immune findings on the full-cohort analysis, present the significance-filtered subset as underpowered and descriptive, and describe all associations as hypothesis-generating.

**Changes:** Methods (Immune infiltration — including a correction of the CIBERSORT execution description to the accurate series-wide wording, see note below; Statistical analysis); Results (immune infiltration paragraph); Discussion (immune paragraph); new S8 Table; S5 Table annotated with the analysis flags.

*Note:* while finalising S9 Dataset we found that the original CIBERSORT run had been executed on the full deposited series (71 samples, including the 34 series samples not used in this study) rather than only on the 37 training samples as previously stated; the Methods description has been corrected accordingly. Because CIBERSORT normalization is performed per sample, all reported values are unchanged.

### R2-6. Pipeline identity does not establish the reliability of LM22 estimates in glomerular tissue

**Reviewer comment:** "Using the same processing pipeline does not in itself demonstrate the reliability of LM22 estimates in glomerular tissue."

**Response:** We agree, and we have strengthened the manuscript at three levels.

(i) **Explicit limitation (expanded).** LM22 is a bulk leukocyte reference matrix: it contains no intrinsic glomerular cell types (podocytes, mesangial cells, glomerular endothelial cells), so its output in glomerular extracts must be read as relative immune-enrichment signals rather than absolute cellular proportions, and residual signal from intrinsic glomerular cells can bias the estimates. The revised Limitations state this directly.

(ii) **Marker-gene concordance check (new analysis).** If the deconvolution estimates are informative, they should correlate with the expression of canonical marker genes measured independently of CIBERSORT. This is the case for the two signals the paper relies on: across the 37 training samples, the CIBERSORT monocyte fraction correlates with the mean expression of CD14, LST1, S100A8, S100A9 and FCN1 (Spearman rho = 0.60, BH-adjusted P = 3.5 x 10-4), and the activated NK cell fraction correlates with NKG7, NCR1, KLRF1, GNLY and PRF1 (rho = 0.48, adjusted P = 0.005). Naive B cell and CD8 T cell estimates — cell types that are rare or absent in glomeruli — show no such concordance (rho = 0.08 and -0.17, n.s.), which is itself informative about where LM22 can and cannot be trusted here. The check is described in the Methods, reported in the Results, and provided per gene in S8 Table.

(iii) **Framing.** All immune findings are now described as hypothesis-generating; confirmation requires cell-type-resolved (single-cell or spatial) profiling of FSGS glomeruli, as stated in the Discussion and Limitations.

**Changes:** Methods; Results; Discussion (caveats); Limitations (LM22 paragraph expanded); S8 Table.

### R2-7. CENPM: external AUC vs non-significant adjusted differential expression

**Reviewer comment:** "Please distinguish CENPM's external AUC value from its non-significant adjusted differential expression result (P = 0.22)."

**Response:** We now distinguish the two results explicitly. In the external cohort, CENPM shows a non-significant expression difference after genome-wide correction (log2FC 0.07; BH-adjusted P = 0.22 from the GEO2R limma model; raw Wilcoxon P = 0.017), while its ROC AUC is 0.720 (95% CI 0.553-0.887). These statistics answer different questions: a differential-expression test asks whether the group means differ and is sensitive to sample size and cohort heterogeneity, whereas the AUC measures ranking-based discrimination, which can be moderate even when the mean shift is small. The revised Results state that CENPM's external evidence is therefore weak and internally discordant — a moderate AUC without a significant adjusted expression difference — and that the AUC should not be interpreted as independent confirmation of CENPM as a candidate biomarker. This reframing is consistent with the fold-wise selection analysis (R2-3), in which CENPM was the less consistently selected of the two genes. Across the Abstract, Results, Discussion and Conclusion, CENPM is now consistently presented as the weaker of the two exploratory candidates, requiring independent confirmation.

**Changes:** Results (external validation); Abstract; Discussion; Conclusion.

### R2-8. Mechanistic, FSGS-vs-MCD and patient-stratification statements are hypothetical

**Reviewer comment:** "Please explicitly state that the claims regarding mechanism, discrimination of FSGS from minimal change disease, and patient stratification are hypothetical."

**Response:** We agree, and we audited the Discussion to make the hypothetical status of all three claim types explicit. (i) Mechanism: the link between AURKA/CENPM and mitotic catastrophe / APC-C-associated programmes is now framed as a hypothesis generated by enrichment and network analyses ("we hypothesise ...; this remains to be tested experimentally"). (ii) FSGS vs MCD: the suggestion that these markers might help separate FSGS from minimal change disease in diagnostically ambiguous biopsies is now explicitly hypothetical — the study design cannot test diagnostic separation, no MCD-only cohort was analysed, and six training samples carry overlapping FSGS/MCD annotations (as disclosed in the Methods and Limitations). (iii) Patient stratification: this is now stated as untestable with the available data, because the public cohorts provide no treatment-response or outcome annotation (Limitations, sixth point); the Discussion now presents it as a question for prospective cohorts. All three passages carry explicit hypothesis language and name the experiments or data that would be required.

**Changes:** Discussion (three passages); Limitations (as cross-references).

### R2-9. Provide analysis scripts and complete software details directly

**Reviewer comment:** "Providing the analysis scripts and full software details directly, rather than only upon request, would help readers verify and reproduce the revised findings."

**Response:** We have done so. The complete analysis pipeline — series-matrix parsing and preprocessing, quality control, top-8,000 variance filtering, WGCNA input preparation, random forest, SVM-RFE, ROC/AUC analysis, and the CIBERSORT post-processing (per-sample tables, FDR analyses, fold-wise stability analysis, marker-gene concordance check) — is provided as Supporting Information (S9 Dataset: the complete analysis pipeline together with a README listing all software and package versions) and is referenced in the Data Availability statement; no request is required. The software and version statement added to the Methods (and repeated in the S9 Dataset README) lists every tool and version: R 4.2.1 (WGCNA via TBtools II v2.439; e1071; randomForest; clusterProfiler; ReactomePA; pROC; ggplot2), Python 3.13 (numpy 2.5.1; scipy 1.18.0; scikit-learn 1.9.0 — used for preprocessing and for the new fold-stability, FDR and concordance analyses), TBtools II v2.439, and CIBERSORT v1.03 (LM22 reference, 1,000 permutations, quantile normalisation) executed via the CIBERSORT web portal. Because the CIBERSORT source code is distributed under a licence that does not permit redistribution, we provide the exact input matrices and per-sample outputs (S5 Table; S9 Dataset) for every CIBERSORT result rather than the source itself. Together with the per-sample supporting tables (S1, S3, S5, S7, S8) and the deposited series matrices (S1 and S2 Datasets), we believe all data and code underlying every figure and statistic in the manuscript are now directly accessible.

**Changes:** Data availability statement rewritten; Methods — software and version statement added; Supporting Information — new S9 Dataset added.

## Closing

We are grateful for the reviewer's persistence on the threshold and overfitting questions: the re-analyses they prompted — threshold-free external reporting, fold-wise selection frequencies, FDR-corrected immune analyses and the marker-gene concordance check — have made the manuscript materially more honest and more reproducible. We hope the revised manuscript is now suitable for publication in PLOS ONE.

Yours sincerely,

Zhang Xikui

The Second Affiliated Hospital of Fujian University of Traditional Chinese Medicine, Fuzhou, Fujian, China

zhangxk2026@outlook.com

On behalf of all authors (Lin Xiaomei, Huang Guofang, Sun Qing, Zhang Xikui)
