# Supplementary material for: Integrated transcriptomic analysis and machine learning identify AURKA and CENPM as candidate biomarkers in focal segmental glomerulosclerosis
Source: PLoS One. 2026 Nov 10;21(11):e0361466. doi: 10.1371/journal.pone.0361466 (PMC13750537; doi:10.1371/journal.pone.0361466)
Supplement: Peer review history

# Peer review: Decision Letter 0

**DOI:** 10.1371/journal.pone.0361466.r001

**Authors:** Claude Prigent

9 Sep 2026

Dear Dr. zhang,

Please respond in detail to each of the comments made by the two reviewers.

For example the reviewer #1 said : "it is hard for me to conceive that such small differences could be use as diagnostic marker in a clinical setting" . Could you explain this point so that your work is accessible to a wider audience?

Also reviewer #2 said : "The enrichment and immune deconvolution results support associations, but do not establish that AURKA and CENPM regulate the proposed pathways or immune infiltration". this is very important, please comment.

Please submit your revised manuscript by Nov 08 2026 11:59PM. If you will need more time than this to complete your revisions, please reply to this message or contact the journal office at ?>plosone@plos.org. When you're ready to submit your revision, log on to https://www.editorialmanager.com/pone/ and select the 'Submissions Needing Revision' folder to locate your manuscript file.

- A letter that responds to each point raised by the academic editor and reviewer(s). You should upload this letter as a separate file labeled 'Response to Reviewers'.
- A marked-up copy of your manuscript that highlights changes made to the original version. You should upload this as a separate file labeled 'Revised Manuscript with Track Changes'.
- An unmarked version of your revised paper without tracked changes. You should upload this as a separate file labeled 'Manuscript'.

If you would like to make changes to your financial disclosure, please include your updated statement in your cover letter. Guidelines for resubmitting your figure files are available below the reviewer comments at the end of this letter.

If applicable, we recommend that you deposit your laboratory protocols in protocols.io to enhance the reproducibility of your results. Protocols.io assigns your protocol its own identifier (DOI) so that it can be cited independently in the future. For instructions see: https://journals.plos.org/plosone/s/submission-guidelines#loc-laboratory-protocols. Additionally, PLOS One offers an option for publishing peer-reviewed Lab Protocol articles, which describe protocols hosted on protocols.io. Read more information on sharing protocols at https://plos.org/protocols?utm_medium=editorial-email&utm_source=authorletters&utm_campaign=protocols.

As the corresponding author, your ORCID iD is verified in the submission system and will appear in the published article. PLOS supports the use of ORCID, and we encourage all coauthors to register for an ORCID iD and use it as well. Please encourage your coauthors to verify their ORCID iD within the submission system before final acceptance, as unverified ORCID iDs will not appear in the published article. *Only*  the individual author can complete the verification step; PLOS staff *cannot*  verify ORCID iDs on behalf of authors.

We look forward to receiving your revised manuscript.

Kind regards,

Claude Prigent

Academic Editor

PLOS One

Journal Requirements:

When submitting your revision, we need you to address these additional requirements.

1. Please ensure that your manuscript meets PLOS ONE’s style requirements, including those for file naming. The PLOS ONE style templates can be found at https://journals.plos.org/plosone/s/file?id=wjVg/PLOSOne_formatting_sample_main_body.pdf and https://journals.plos.org/plosone/s/file?id=ba62/PLOSOne_formatting_sample_title_authors_affiliations.pdf

2. We note that the grant information you provided in the ‘Funding Information’ and ‘Financial Disclosure’ sections do not match.

When you resubmit, please ensure that you provide the correct grant numbers for the awards you received for your study in the ‘Funding Information’ section.

3. Thank you for stating the following financial disclosure:

[The author(s) received no specific funding for this work.].

At this time, please address the following queries:

a) Please clarify the sources of funding (financial or material support) for your study. List the grants or organizations that supported your study, including funding received from your institution.

b) State what role the funders took in the study. If the funders had no role in your study, please state: “The funders had no role in study design, data collection and analysis, decision to publish, or preparation of the manuscript.”

c) If any authors received a salary from any of your funders, please state which authors and which funders.

d) If you did not receive any funding for this study, please state: “The authors received no specific funding for this work.”

Please include your amended statements within your cover letter; we will change the online submission form on your behalf.

4. In the online submission form, you indicated that [Further inquiries can be directed to the corresponding author.].

All PLOS journals now require all data underlying the findings described in their manuscript to be freely available to other researchers, either 1. In a public repository, 2. Within the manuscript itself, or 3. Uploaded as supplementary information.

This policy applies to all data except where public deposition would breach compliance with the protocol approved by your research ethics board. If your data cannot be made publicly available for ethical or legal reasons (e.g., public availability would compromise patient privacy), please explain your reasons on resubmission and your exemption request will be escalated for approval.

5. Please include a separate caption for each figure in your manuscript.

6. Please include captions for your Supporting Information files at the end of your manuscript, and update any in-text citations to match accordingly. Please see our Supporting Information guidelines for more information: http://journals.plos.org/plosone/s/supporting-information.

7. We are unable to open your Supporting Information file [._GSE104948sample.xlsx]. Please kindly revise as necessary and re-upload.

If the reviewer comments include a recommendation to cite specific previously published works, please review and evaluate these publications to determine whether they are relevant and should be cited. There is no requirement to cite these works unless the editor has indicated otherwise.

[Note: HTML markup is below. Please do not edit.]

Reviewers' comments:

Reviewer’s Responses to Questions

**Comments to the Author**

1. Is the manuscript technically sound, and do the data support the conclusions?

Reviewer #1: Partly

Reviewer #2: Partly

**********

2. Has the statistical analysis been performed appropriately and rigorously?

Reviewer #1: I Don't Know

Reviewer #2: Yes

**********

3. Have the authors made all data underlying the findings in their manuscript fully available?

The PLOS Data policy

Reviewer #1: Yes

Reviewer #2: Yes

**********

4. Is the manuscript presented in an intelligible fashion and written in standard English?

Reviewer #1: Yes

Reviewer #2: Yes

**********

Reviewer #1: Focal segmental glomerulosclerosis (FSGS) is a renal condition that accounts for a large number of nephropathies in both adults and children. PSGS is typically diagnosed after histological analyses of kidney biopsies, underlining the urgent need for less- to non-invasive diagnostic methods, such as biomarkers.

To fill this gap, Lin Xiaomei and colleagues made use of publicly available FSGS patient expression dataset. Through a variety of computational approaches, the authors identified two potential mRNA biomarkers, i.e., Aurora A and CENP-M. Aurora A is a S/T kinase that plays essential roles in many aspects of cell biology, such as cell cycle control and progression, gene expression, centrosome regulation, spindle assembly and dynamics. Somewhat similar, CENP-M is part of pericentromeric heterochromatin that is tightly linked to kinetochore assembly and function, and is therefore also key to proper cell cycle progression and cell division. As discussed by the authors, such impaired functions may well account for at least part of the disease manifestations.

Major comments:

1- I appreciated that the authors first trained their models on first datasets and then assessed their efficiency/efficacy on distinct ones.

2- However, the gene expression differences revealed by the authors 'analyses are very small (around 5 to 7 % of control samples, if I am correct). If statistically significant, it is hard for me to conceive that such small differences could be use as diagnostic marker in a clinical setting. In addition, these tiny differences concern mRNA levels, and may have no impact on corresponding protein levels, even more so on their cellular functions. Both these points should at least be discussed.

3- In the discussion, the authors indicate that Aurora A is "highly expressed in the FSGS cohort". This is not the case based on provided data.

4- As the authors have used PSGS biopsy samples, I wonder how the identification of these two markers could represent an improvement toward histopathological examination. Have the authors analysed patient blood samples?

5- The authors rightly mentioned therapy-resistant patients. What is the status of patient sample used in the study? Can Aurora A and/or CENPM help to stratify patients?

To conclude, Aurora A and CENP-M might indeed represent interesting FSGS biomarkers but the very small differences observed between patient cohort and healthy population render this unlikely in my view, which also questions the interest of the presented work to PLoS readers.

Reviewer #2: The manuscript addresses a relevant question in focal segmental glomerulosclerosis and brings together established transcriptomic, network, and machine learning approaches to identify AURKA and CENPM as candidate biomarkers. The use of an additional dataset and the connection to cell cycle biology are useful strengths. Comments:

- The GEO record for GSE104948 explicitly states that samples were previously included in GSE47183, which is used here for external validation. Please provide the exact sample identifiers, verify patient and specimen independence, and repeat validation using a genuinely independent cohort if overlap is present.

- The small sample sizes also make the feature selection procedure and reported performance vulnerable to overfitting. Please clarify whether all preprocessing, gene filtering, WGCNA, random forest, and SVM-RFE selection were performed within the training data, and reconcile the Methods and Results discrepancy between 8000 and 12074 genes, as well as the random forest importance threshold and Figure 3. The exact external AUCs, confidence intervals, and classification performance should be reported rather than described only as exceeding 0.8.

- Since the study analyzes renal tissue expression rather than blood or urine, the findings should be framed as exploratory tissue biomarkers, not evidence of a non-invasive diagnostic test or established clinical applicability.

- The title also promises multi-omics integration, whereas the presented analysis appears to rely on transcriptomic data with downstream computational analyses.

- The enrichment and immune deconvolution results support associations, but do not establish that AURKA and CENPM regulate the proposed pathways or immune infiltration. Please discuss possible cell composition effects, the suitability of LM22 for glomerular tissue, and the need for independent experimental validation.

- A clearer comparison with prior FSGS biomarker studies and a complete, reproducible account of the analytical workflow would considerably strengthen the manuscript.

**********

what does this mean?). If published, this will include your full peer review and any attached files.

If you choose “no”, your identity will remain anonymous but your review may still be made public.

**Do you want your identity to be public for this peer review?**  For information about this choice, including consent withdrawal, please see our Privacy Policy

Reviewer #1: **Yes:**  Erwan Watrin

Reviewer #2: No

**********

[NOTE: If reviewer comments were submitted as an attachment file, they will be attached to this email and accessible via the submission site. Please log into your account, locate the manuscript record, and check for the action link "View Attachments". If this link does not appear, there are no attachment files.]

To ensure your figures meet our technical requirements, please review our figure guidelines: https://journals.plos.org/plosone/s/figures

You may also use PLOS’s free figure tool, NAAS, to help you prepare publication quality figures: https://journals.plos.org/plosone/s/figures#loc-tools-for-figure-preparation.

NAAS will assess whether your figures meet our technical requirements by comparing each figure against our figure specifications.

# Author comment: Author response to Decision Letter 1

**DOI:** 10.1371/journal.pone.0361466.r002

21 Sep 2026

Response to Reviewers

Manuscript title: Integrated transcriptomic analysis and machine learning identify AURKA and CENPM as candidate biomarkers in focal segmental glomerulosclerosis

Journal: PLOS ONE

Academic Editor: Prof. Claude Prigent

Manuscript ID: PONE-D-26-33048

Authors: Lin Xiaomei¹, Huang Guofang¹,², Sun Qing¹,², Zhang Xikui³*

¹Fujian University of Traditional Chinese Medicine, Fuzhou, Fujian, China

²Rehabilitation Hospital Affiliated to Fujian University of Traditional Chinese Medicine, Fuzhou, Fujian, China

³The Second Affiliated Hospital of Fujian University of Traditional Chinese Medicine, Fuzhou, Fujian, China

*Corresponding author: Zhang Xikui (zhangxk2026@outlook.com)

Dear Prof. Prigent,

We thank you and the two reviewers for the careful and constructive evaluation of our manuscript. The comments identified several substantial issues — in particular the independence of the validation cohort, the risk of overfitting in a small training set, and the framing of our findings — and addressing them has materially improved the work. We have revised the manuscript accordingly; all changes are highlighted in the marked-up copy, and a clean version is provided.

Three changes are central to this revision:

1. The external validation cohort has been replaced, and the validation is now genuinely independent. Following Reviewer 2’s observation, we re-examined the provenance of the datasets. GSE104948 and GSE47183 are indeed derived from the same patient series (the European Renal cDNA Bank, ERCB): the GSE104948 record states that its samples were "included under previous GEO submissions — GSE47183 (+GSE32591)". On re-annotation, the two series share seven patients (FSGS915/917/918/927 and TN995/996/997), and 12 of the 27 samples (44%) of the GSE47183 "validation" set come from patients already present in the training set; GSE47183 additionally contains technical replicates of the same biopsy (its 14 "controls" represent only five patients). We therefore discarded GSE47183 altogether and repeated external validation in GSE129973 (Nanjing University; 20 FSGS vs 20 controls; glomerular tissue), a cohort from a different institution and biobank with no patient overlap with the training set. As a consequence, the reported external performance is lower but honest: AURKA AUC 0.797 (95% CI 0.661–0.934), CENPM AUC 0.720 (95% CI 0.553–0.887), versus 0.907/0.830 in the overlapping cohort.

2. The study has been repositioned. The title, abstract, results, discussion and conclusion now present AURKA and CENPM as exploratory tissue-based candidate biomarkers and hypothesis-generating findings, not as established diagnostic markers or non-invasive tests.

3. The training cohort is now fully disclosed. Six of the 16 training FSGS samples carry overlapping FSGS/MCD annotations in the original ERCB series; this is now stated in the Methods and in the Limitations, with a sensitivity analysis provided as S4 Table.

We are grateful for the opportunity to revise the manuscript and hope it is now suitable for publication in PLOS ONE.

Part A. Editorial requirements

A1. Formatting and file naming

Editor: Please ensure that your manuscript complies with the PLOS ONE format requirements, including the file naming rules.

Response. The manuscript has been checked against the PLOS ONE formatting templates, and file naming has been standardised: main figures as `Fig1.tif`–`Fig7.tif`, Supporting Information as `S1_Table.xlsx`–`S6_Table.xlsx` and `S1_Fig.tif`–`S2_Fig.tif`. Each figure now has its own caption in the manuscript text, placed directly after the paragraph in which the figure is first cited, as required by the PLOS ONE template; the captions are not embedded in the image files, in line with PLOS ONE policy.

Changes in the manuscript: Figure section rebuilt; all figure legends rewritten (one per figure, each ending with a brief Statistics sentence).

A2 and A3. Funding information and financial disclosure

Editor: We noticed that the funding information you provided in the "Funding Information" and "Financial Disclosure" sections does not match. Please ensure that you provide the correct grant number(s) for awards that you received for this study. Please also answer the following questions (a–d).

Response. We apologise for the inconsistency in the original submission. The study received no specific funding, and the two entries have now been made consistent.

(a) Sources of funding (including grants and material support): the authors received no specific funding for this work. No funds or material support were received from any funding agency or organisation, including the authors' institution.

(b) Role of the funder: not applicable — there was no funding source, and therefore no funder had any role in study design, data collection and analysis, decision to publish, or preparation of the manuscript.

(c) Payments to authors from any funder: none. No author has received any salary, payment or other benefit from any funding body.

(d) Explicit statement: The authors received no specific funding for this work.

Changes in the manuscript: the Funding section now reads "The authors received no specific funding for this work." We kindly ask the editorial office to update the corresponding fields of the submission system accordingly.

A4. Data availability

Editor: In the online submission form you stated "[Further inquiries can be directed to the corresponding author]". All PLOS journals require that all data underlying the findings be made freely available.

Response. We have replaced the data availability statement with an explicit pointer to the public repositories: the training cohort is available in GEO under GSE104948 and the independent validation cohort under GSE129973. In addition, per-sample data underlying every summary statistic reported in the manuscript are provided as Supporting Information (per-sample quality-control statistics as S3 Table, per-sample CIBERSORT deconvolution results as S5 Table, and per-sample sample-level information as S1 Table).

Changes in the manuscript: Data availability statement rewritten (Section 10).

A5. A caption for each figure

Editor: Please add a caption for each figure in the manuscript.

Response. We confirm that every main figure now has its own caption in the manuscript, with each caption placed directly after the paragraph in which the figure is first cited; the figure files themselves contain no caption text, in line with PLOS ONE policy. All legends were rewritten from scratch to match the actual panel structure of the figures, and each statistical legend ends with a short Statistics sentence describing the test used.

Changes in the manuscript: Fig 1–Fig 7 legends (each placed directly after its first citation in the text).

A6. Titles of supporting information files and corresponding in-text citations

Editor: Please add the titles of the Supporting Information files at the end of the manuscript and update the in-text citations accordingly.

Response. The Supporting Information section now lists all eight files with full titles, in numerical order — Tables S1–S6 followed by Figures S1–S2 — and every item is now cited in the main text: S1 (sample information and cohort independence, Methods → Data Collection; ), S2 (the 39 candidate genes, Results), S3 and Figures S1–S2 (quality control, Methods → Data Collection), S4 (sensitivity analysis, Limitations), S5 (CIBERSORT per-sample results, Methods → Immune infiltration, and the Fig 6 legend), S6 (robustness of feature selection, Results → Feature selection).

Changes in the manuscript: Supporting Information section reorganised; in-text citations added in the Methods and Results.

A7. The supplementary file that could not be opened

Editor: We were unable to open your supplementary file [._GSE104948sample.xlsx]. Please modify and re-upload as appropriate.

Response. Thank you for flagging this. The file we were unable to open, `._GSE104948sample.xlsx`, was not a data file: it is a macOS resource-fork artefact (AppleDouble) that was inadvertently included when the original supplementary file was uploaded — a known issue when files are packaged on macOS. The artefact has been removed. The data file itself (`GSE104948sample.xlsx`) contained only a sample list (group assignment and GEO accession for the 37 training samples); this information is now provided in a substantially expanded form as S1 Table, which lists the GEO accession identifier, the original ERCB annotation, the analysis group, the diagnosis/control type and the sensitivity-analysis status for every sample of both cohorts (37 training + 40 validation samples). All supplementary files have been verified to open correctly.

Changes in the manuscript: Supporting Information section (eight files, PLOS-compliant naming); the previous file is no longer part of the submission.

A8 and A9. The two points highlighted in the editorial letter

Editor: Reviewer 1 said "I find it hard to imagine that such a small difference could be used as a clinical diagnostic marker." Reviewer 2 pointed out that "the enrichment and immune deconvolution results support correlation but do not confirm that AURKA and CENPM regulate the proposed pathways or immune infiltration."

Response. Both points are addressed in detail in our responses to Reviewer 1, comment 2 and Reviewer 2, comment 5, respectively. In brief: (i) we now explicitly acknowledge the modest effect size and report the honest, lower discrimination obtained in the independent cohort, and we reposition the genes as exploratory tissue-based candidates whose value lies in the convergence of independent analytical lines rather than in single-gene effect size; (ii) all enrichment and immune-deconvolution results are now described as association-only evidence, with dedicated statements in the Methods, the Results and the Discussion that these analyses "do not establish a regulatory relationship".

Part B. Reviewer 1

R1-1. Training and external evaluation in separate datasets

Reviewer 1 comment 1: "I appreciate that the authors first trained their model on an initial dataset and then evaluated its efficiency/efficacy in a different dataset."

Response. We thank the reviewer for this comment. In response to Reviewer 2 (comment 1), we have taken this principle further: the external evaluation is now performed in a cohort that is not only a different dataset but, unlike the original GSE47183 series, also independent at the patient level (different institution and biobank, no overlapping patients; see Part C, comment 1).

Changes in the manuscript: External validation section; Tables and Fig 7 updated to GSE129973.

R1-2. The magnitude of the expression difference and the mRNA-to-protein gap

Reviewer 1 comment 2: "The gene expression differences revealed by the authors' analysis are very small (about 5% to 7% of controls). Even if statistically significant, I find it hard to imagine that such a small difference could be used as a clinical diagnostic marker. Moreover, these small differences relate to mRNA levels and may have no effect on the corresponding protein levels, let alone on cellular function. These two points should at least be discussed."

Response. We fully agree that both points deserve explicit discussion, and we have addressed them in the revised manuscript.

(i) Effect size and diagnostic performance. We now state the effect sizes explicitly (training cohort: log2FC 0.47 for AURKA and 0.40 for CENPM, i.e. approximately 1.3-fold; independent cohort: 0.18 and 0.07) and report the corresponding discrimination honestly. Crucially, when the overlapping validation set was replaced by the truly independent cohort, the external performance dropped from AUC 0.907/0.830 to 0.797 (95% CI 0.661–0.934) / 0.720 (95% CI 0.553–0.887) — at the Youden-optimal cut-off this corresponds to 90.0% sensitivity with only 60.0% specificity for AURKA and 55.0% for CENPM (accuracy 75.0% and 72.5%), which is clearly insufficient for a diagnostic test. We have therefore removed any claim that these genes constitute a diagnostic biomarker and instead present them as exploratory tissue-based candidates. We consider it more valuable to report the lower, honest performance in a genuinely independent cohort than to retain inflated estimates contaminated by sample overlap. To make this contrast explicit and to avoid any impression that the training-cohort figures stand alone, the Results now describe the training-cohort finding as "high discriminative performance in the training cohort, in contrast to the lower performance in the independent validation cohort".

(ii) mRNA versus protein. We have added a dedicated limitation stating that the study is based on transcriptomic data alone and that protein-level and functional validation was not performed, and we have added an explicit note on the interpretation of mRNA-level findings: a 1.3-fold transcript change need not translate into a proportional change in protein abundance or activity. To provide at least indirect protein-level context, we consulted the Human Protein Atlas: both AURKA (antibodies HPA002636/CAB001454) and CENPM (antibody HPA042404) have antibody-based evidence at the protein level in human kidney, with low staining in tubular cells and consensus RNA expression of 2.3 nTPM and 2.1 NX, respectively; notably, glomerular staining was not detected in healthy tissue, which is the expected baseline for cell-cycle-associated genes in quiescent glomeruli. We are explicit that this is contextual support and not a substitute for disease-state, glomerulus-resolved protein validation, which remains future work.

Changes in the manuscript: Results (ROC section) — training-cohort wording revised ("excellent diagnostic performance" → "high discriminative performance in the training cohort, in contrast to the lower performance in the independent validation cohort"); Results (external validation) — effect sizes, AUCs with confidence intervals and classification performance added; Limitations, third and fourth points — modest effect size, mRNA-to-protein caveat and HPA evidence.

R1-3. The description of AURKA expression

Reviewer 1 comment 3: "In the discussion, the authors state that Aurora A is 'highly expressed in the FSGS cohort'. According to the data provided, this is not the case."

Response. The reviewer is correct, and we apologise for this overstatement. The sentence now reads that AURKA was "moderately upregulated" in the FSGS cohort, which matches the observed effect size. We have also searched the entire manuscript for comparable expressions and replaced them with wording consistent with the data.

Changes in the manuscript: Discussion (AURKA paragraph): "highly expressed" → "moderately upregulated".

R1-4. How tissue-derived markers relate to histopathology, and whether blood was analysed

Reviewer 1 comment 4: "Since the authors use FSGS biopsy samples, I wonder how the identification of these two markers could improve histopathological examination. Have the authors analysed blood samples from the patients?"

Response. Two separate questions are involved.

(i) Blood samples. No — no blood (or urine) samples were available for the patients in the public datasets used here. Both genes are therefore explicitly not proposed as non-invasive blood- or urine-based markers. This is now stated as a limitation.

(ii) How such markers could add value on top of histopathology. We have added a dedicated passage to the Discussion clarifying the intended positioning. Because FSGS is defined histologically, markers measured in the same biopsy specimen are not intended to replace histological assessment. Their added value lies in the molecular dimension that light microscopy alone cannot provide: helping to separate FSGS from minimal change disease in diagnostically ambiguous biopsies, stratifying patients by the activity of cell-cycle and mitotic programmes, and nominating candidate therapeutic targets such as AURKA. We also note explicitly that prospective studies linking such molecular readouts to treatment response are required before clinical utility can be claimed.

Changes in the manuscript: Limitations, sixth point (blood/urine samples not available); Discussion (comparison with previously reported biomarkers) — new passage on positioning relative to histopathology.

R1-5. Patient sample status and patient stratification

Reviewer 1 comment 5: "The authors correctly mention treatment-refractory patients. What is the status of the patient samples used in this study? Could Aurora A and/or CENP-M be used for patient stratification?"

Response. The public datasets used here (GSE104948 and GSE129973) provide only the diagnosis (FSGS versus control) with limited clinical annotation; treatment response, disease stage and follow-up outcome are not available for most samples. We therefore cannot determine whether AURKA or CENPM expression differs between treatment-responsive and treatment-resistant patients, or whether they could contribute to patient stratification. Rather than speculate, we state this as an explicit limitation and point out that answering the question requires prospective cohorts with matched biospecimens and treatment-outcome data.

Changes in the manuscript: Limitations, sixth point.

R1-6 (overall assessment). Significance for the readership

Reviewer 1 (concluding remark): "Aurora A and CENP-M may indeed be promising FSGS biomarkers, but the differences observed between patient cohorts and healthy individuals are very small, which in my view makes this possibility unlikely, and this also raises questions about the significance of the paper for PLOS readers."

Response. We have taken this concern seriously and have restructured the framing of the paper accordingly. The study is now presented explicitly as hypothesis-generating work whose contribution is not a diagnostic test but: (i) the convergent identification of two cell-cycle genes by three independent analytical routes (WGCNA module membership, random forest, SVM-RFE, and PPI-based MCC) — the intersection being robust across all 24 seed × threshold configurations tested; (ii) an independent-cohort expression profile with an honest, moderate discriminative performance; and (iii) a mechanistic link to cell-cycle and mitotic programmes in a disease whose podocyte loss is known to involve mitotic catastrophe. We hope this repositioning, together with the explicit limitations, makes the value of the work clear to the readership — as a starting point for protein-level and functional follow-up rather than as a clinical claim.

Changes in the manuscript: Title; Abstract (Conclusion); Results (final paragraph); Discussion; Conclusion — consistent repositioning as exploratory tissue-based candidate biomarkers.

Part C. Reviewer 2

R2-1. Sample overlap between the training and validation cohorts

Reviewer 2 comment 1: "The GEO record for GSE104948 clearly states that the samples were previously included in GSE47183, which is used here for external validation. Please provide the exact sample identifiers, verify the independence of patients and samples, and if there is overlap, repeat the validation using a truly independent cohort."

Response. The reviewer is entirely correct, and the problem proved to be more extensive than the GEO record alone suggests. Our verification found that:

- The GSE104948 series matrix states explicitly that its samples were "included under previous GEO submissions — GSE47183 (+GSE32591)"; both are ERCB collections, and GSE104948 represents a re-annotation of the same biological material (GSM 2810xxx vs GSM 1146xxx).

- At the patient level, the training and "validation" subsets share seven patients (FSGS915, FSGS917, FSGS918, FSGS927, TN995, TN996, TN997). Twelve of the 27 GSE47183 samples (44%) therefore come from patients already used for training.

- GSE47183 additionally contains technical replicates of the same biopsy: its 14 samples labelled as controls correspond to only five patients (TN2 ×3, TN4 ×3, TN995 ×2, TN996 ×4, TN997 ×4). Of its nine "new" FSGS samples, eight also belong to the GSE104948 series.

Accordingly, we have discarded GSE47183 in its entirety and re-performed external validation in GSE129973 (Nanjing University Second Affiliated Hospital; 20 FSGS vs 20 controls; glomerular tissue), a cohort from a different institution and biobank that shares no patients with GSE104948.

Original validation set (GSE47183) New validation set (GSE129973)

Source ERCB (same biobank as training set) Nanjing University (independent)

Patient overlap with training set 7 shared patients; internal technical replicates None

Samples 13 FSGS + 14 controls (with replicates) 20 FSGS + 20 controls

AURKA AUC 0.907; adj. P = 0.060 AUC 0.797; adj. P = 0.012

CENPM AUC 0.830; adj. P = 0.204 AUC 0.720; adj. P = 0.22

Exact sample identifiers, together with the original ERCB annotation and the analysis group for every sample, are provided in S1 Table (S1a: 37 training samples; S1b: 40 validation samples).

We chose to report the honest, lower performance in a truly independent cohort rather than retain inflated estimates contaminated by sample overlap.

Changes in the manuscript: Methods (Data Collection) — cohort description, statement that no patient overlap exists, and citation of S1 Table; Results (external validation) — all values re-computed; Fig 7 replaced with the GSE129973 analysis; Abstract, Discussion and Limitations updated throughout.

R2-2. Risk of overfitting and the reporting of performance

Reviewer 2 comment 2: "The small sample size also makes the feature-selection process and the reported performance prone to overfitting. Please clarify whether all preprocessing, gene filtering, WGCNA, random forest and SVM-RFE selection were performed in the training data, and explain the discrepancy between the 8,000 and 12,074 genes in the Methods and Results, as well as the discrepancy between the random-forest importance threshold and Fig 3. Exact external AUC values, confidence intervals and classification performance should be reported rather than just described as 'above 0.8'."

Response. We address the four points in turn.

(a) Independence of all modelling steps. We have made this explicit: all preprocessing, gene filtering, WGCNA network construction and feature selection (random forest, SVM-RFE and cytoHubba MCC) were performed exclusively on the training cohort; the independent validation cohort was processed separately and used only for evaluation. For completeness, the preprocessing and quality-control steps were as follows: quality control of both cohorts comprised inspection of per-sample expression distributions, principal component analysis and hierarchical clustering, and no sample was excluded (Figures S1–S2; per-sample statistics in S3 Table); preprocessing used the depositor-provided, log2-scale gene-level matrix (Human Entrez Gene ID custom CDF version 19; 12,074 genes), with Entrez identifiers annotated to gene symbols and duplicates collapsed (12,045 genes); gene filtering/selection (the top 8,000 most variable genes) and all downstream modelling were then performed exclusively within the training cohort, while the validation cohort (GSE129973, HTA 2.0) was processed separately. All of these steps are now described explicitly in the Methods (Data Collection and Preprocessing; WGCNA).

(b) 8,000 versus 12,074 genes. These numbers refer to two different steps and are not in conflict: 12,074 genes were retained after preprocessing of GSE104948, and the top 8,000 most variable genes (of these 12,074) were used as input for WGCNA network construction. The wording is now identical in the Methods and the Results ("the top 8,000 most variable genes (out of 12,074 preprocessed genes)").

(c) Random-forest threshold and Fig 3. The random-forest model used 500 trees and genes with a Mean Decrease Gini (MDG) > 1.0 were retained, yielding five candidate genes; this is now stated both in the Methods and in the Results, and Fig 3A is accompanied by a legend describing exactly what is plotted and what the threshold represents. To address the overfitting concern directly, we performed a systematic robustness analysis: across 8 random seeds × 3 MDG thresholds (1.0, 0.9, 0.8) = 24 configurations, the intersection of the three methods (random forest, SVM-RFE, cytoHubba MCC) was AURKA and CENPM in every single configuration. This is now reported in the Results and provided in full as S6 Table.

(d) Exact external performance. We now report the exact values with confidence intervals and classification performance for the independent cohort: AURKA AUC 0.797 (95% CI 0.661–0.934), and CENPM AUC 0.720 (95% CI 0.553–0.887). At the Youden-optimal cut-off, AURKA achieved 90.0% sensitivity and 60.0% specificity (overall accuracy 75.0%), and CENPM 90.0% sensitivity and 55.0% specificity (accuracy 72.5%). For completeness, the corresponding training-cohort values are AURKA AUC 0.949 (0.883–1.000) and CENPM AUC 0.943 (0.875–1.000). We deliberately do not describe performance as "above 0.8" anywhere.

Changes in the manuscript: Methods (Data Collection and Preprocessing — quality control and preprocessing now explicit; Screening of Core Genes; Statistical analysis; Immune infiltration) — statements of training-only procedures, of the preprocessing steps and of the Youden-based metrics; Results (WGCNA; Feature selection; External validation) — unified gene counts, five-gene RF threshold, 24-configuration robustness result, exact AUCs with CIs and classification performance; Fig 3 and Fig 7 legends; S6 Table.

R2-3. Positioning as exploratory tissue biomarkers

Reviewer 2 comment 3: "Because the study analyses kidney tissue expression rather than blood or urine, the findings should be defined as exploratory tissue biomarkers rather than a non-invasive diagnostic test or established evidence for clinical application."

Response. We agree and have repositioned the study throughout. The phrase "exploratory tissue-based candidate biomarkers" now appears in the Abstract (Conclusion), the Results and the Discussion; all statements implying established clinical utility or non-invasive testing have been removed. As noted above, we also report explicitly that the genes cannot serve as blood- or urine-based markers with the data available.

Changes in the manuscript: Title (see R2-4); Abstract; Results (final paragraph of the validation section); Discussion; Conclusion.

R2-4. The title and the "multi-omics" claim

Reviewer 2 comment 4: "The title promises multi-omics integration, but the analyses presented appear to rely on transcriptomic data and downstream computational analysis."

Response. The reviewer is right: our data are transcriptomic, and downstream enrichment, network and deconvolution analyses do not constitute additional omics layers. The title has been changed accordingly.

Changes in the manuscript: Title — now "Integrated transcriptomic analysis and machine learning identify AURKA and CENPM as candidate biomarkers in focal segmental glomerulosclerosis" (sentence case per the PLOS ONE title-page template; previously "Multi-Omics … Core Biomarkers" in title case).

R2-5. Enrichment and deconvolution results support association, not regulation

Reviewer 2 comment 5: "The enrichment and immune deconvolution results support association but do not confirm that AURKA and CENPM regulate the proposed pathways or immune infiltration. Please discuss possible cell-composition effects, the applicability of LM22 to glomerular tissue, and the need for independent experimental validation."

Response. We agree entirely, and we have addressed this at three levels.

(i) Wording. Statements about enrichment and immune infiltration have been changed from regulatory language (for example, "further expand the regulatory landscape of APC/C-associated pathways") to association language ("further support the involvement of … in APC/C-associated pathways"), and the Results now state explicitly that the observed associations "do not imply causality".

(ii) Methodological statements. The Methods now state that these analyses "quantify association only and do not establish a regulatory relationship", and the correlation analyses underlying Fig 6B are described in full (cell–cell associations by Pearson correlation; gene–cell associations by Spearman rank correlation, with block size/link width encoding association strength and link colour encoding the significance class).

(iii) Two specific caveats requested by the reviewer.

- Cell-composition effects. CIBERSORT estimates relative cell fractions from bulk tissue expression, so differences between groups may partly reflect a shift in tissue cell composition rather than a change in per-cell expression of AURKA/CENPM; because both genes are expressed at low levels in quiescent glomeruli, a small number of infiltrating cells could disproportionately influence their apparent correlation with immune cell fractions.

- Applicability of LM22 to kidney. The LM22 signature matrix was derived mainly from peripheral blood leukocytes and therefore does not fully represent the immune cell populations of the kidney; glomerular tissue contains relatively few resident leukocytes, and several cell types prominent in blood are inferred from a limited number of marker genes. Relative differences remain internally comparable because all samples were processed with an identical pipeline and the same signature matrix. We also note that CD4+ naïve T cells and resting eosinophils were not detected in these samples.

- Need for independent experimental validation. We now state explicitly that establishing that AURKA and CENPM actually regulate the pathways or immune profiles with which they correlate will require independent experimental work, including detection of the two proteins in situ in FSGS biopsies, cell-type-resolved profiling, and functional experiments in podocytes or glomerular endothelial cells.

Changes in the manuscript: Methods (Statistical analysis; Immune infiltration); Results (immune infiltration paragraph); Discussion (immune paragraph; two caveats paragraph; comparison with previously reported biomarkers).

R2-6. Comparison with previous biomarkers and a complete, reproducible workflow

Reviewer 2 comment 6: "The manuscript would be substantially strengthened by a clearer comparison with previous FSGS biomarker studies and by a complete, reproducible description of the analytical workflow."

Response. We have added both.

(i) Comparison with previously reported biomarkers. A dedicated passage now compares our findings with circulating and urinary candidates — soluble urokinase-type plasminogen activator receptor (suPAR), anti-nephrin autoantibodies, and urinary NGAL and KIM-1 — noting that those markers have the advantage of being measurable non-invasively but that their specificity for FSGS, particularly in distinguishing FSGS from other proteinuric glomerulopathies, remains debated. We state that AURKA and CENPM were derived from glomerular tissue and therefore cannot be proposed as non-invasive tests, and that they provide molecular candidates for tissue-level characterisation and mechanistic follow-up. We also highlight that, unlike single-marker approaches, they were selected within an unbiased multi-method framework and validated in an independent glomerular cohort.

(ii) Reproducible workflow. The Methods now specify the key parameters of every step (soft-thresholding power 8 with R² = 0.85; minimum module size 30; module membership and gene significance > 0.5; 500 decision trees with MDG > 1.0; five-fold cross-validation for SVM-RFE; top-20 MCC genes; 1,000 permutations and quantile normalisation for CIBERSORT), together with the R packages used (WGCNA in TBtools II; e1071; randomForest; clusterProfiler; ReactomePA; pROC; ggplot2; R version 4.2.1). The Methods also describe the preprocessing and quality-control stage with the same specificity: per-sample expression-distribution inspection, PCA and hierarchical clustering (no sample excluded; Figures S1–S2 and Table S3); the depositor-provided log2-scale gene-level matrix (Entrez CDF version 19; 12,074 genes), with Entrez identifiers annotated to gene symbols and duplicates collapsed (12,045 genes); and variance-based selection of the top 8,000 most variable genes. In addition, the Data availability statement now notes that all analysis scripts (preprocessing, quality control, WGCNA, feature selection, ROC analysis and immune-infiltration analysis) are available from the corresponding author upon reasonable request.

Changes in the manuscript: Discussion (comparison with previously reported biomarkers — new passage); Methods throughout (including Data Collection and Preprocessing: quality control and gene filtering); Data availability statement.

Part D. Additional corrections made during revision

While preparing this revision we identified and corrected several further issues; we report them here for full transparency.

D1. Percentage of significant CIBERSORT deconvolutions. The Methods previously left the number of retained samples unspecific. CIBERSORT was run on all 37 training samples (1,000 permutations, quantile normalisation); significant deconvolution (P < 0.05) was obtained in 12 of 37 samples (8 FSGS and 4 controls). Per-sample values are provided in S5 Table.

D2. Terminology. "GSEA" and "ssGSEA" were used interchangeably in the original text; the analysis actually performed was GSEA (clusterProfiler, gene sets from the curated pathway (CP) subcollection of the MSigDB C2 collection, genes ranked by log2 fold change). The terminology has been unified, and the description of the gene-set source corrected.

D3. Statistical label in Fig 6B. During revision we re-computed the statistics underlying Fig 6B from the original per-sample data and found that the links between the hub genes and immune cell fractions were generated with Spearman rank correlation (the reported r and P values match Spearman exactly for all 40 links), whereas the embedded legend labelled them as "Mantel". The embedded legend has been corrected to "Spearman’s p/r", and the corresponding Methods, Results and figure legend text now describe the two types of association correctly (cell–cell: Pearson; gene–cell: Spearman). The heat-map colour scale ("Pearson’s r") was verified as correct and left unchanged.

D4. Training-cohort composition. Six of the 16 training FSGS samples carry overlapping FSGS/MCD annotations in the original ERCB series (GSM2810777–GSM2810782). This is now disclosed in the Methods and in the Limitations. A sensitivity analysis (S4 Table) shows that after excluding these six samples, AURKA remained strongly upregulated (log2FC 0.513, AUC 0.986) and CENPM was essentially unchanged (log2FC 0.356, AUC 0.933); the inclusion of these borderline cases therefore introduced a conservative bias rather than inflating the signal. The remaining samples of the series (diabetic nephropathy, pure MCD, and RPGN) were not included in any analysis.

D5. S6 Table added. A new supporting table reports the robustness of the three-method intersection across 8 random seeds × 3 MDG thresholds.

D6. How the two panels of Fig 4 are defined. The manuscript previously described Fig 4A and 4B as gene sets "associated with AURKA" and "associated with CENPM", which implied that a separate GSEA had been run for each gene. This was not the case: a single GSEA was performed on genes ranked by the FSGS-versus-control log2 fold change. We have therefore re-written the Methods, Results, Discussion and the Fig 4 legend to state this explicitly, and the two panels are now described as two functional groups of enriched pathways, with the link to the hub genes specified precisely: AURKA is a leading-edge (core-enriched) gene of the two APC/C gene sets shown in panel A, and CENPM is a leading-edge gene of all four gene sets shown in panel B. We have also relabelled the two panels of Fig 4 to describe the functional groups (‘Translation / APC/C programmes’ and ‘M phase / checkpoint programmes’) rather than a single gene, so that the figure no longer implies a per-gene analysis. No data were re-analysed and the enrichment plots themselves are unchanged.

We thank the editor and both reviewers again for their time and for comments that have substantially improved the rigour and the clarity of the manuscript. We would be glad to provide any further information or analyses that may be required.

Yours sincerely,

Zhang Xikui

The Second Affiliated Hospital of Fujian University of Traditional Chinese Medicine, Fuzhou, Fujian, China

zhangxk2026@outlook.com

On behalf of all authors (Lin Xiaomei, Huang Guofang, Sun Qing, Zhang Xikui)

Attachment Submitted filename: Response to Reviewers.docx

# Peer review: Decision Letter 1

**DOI:** 10.1371/journal.pone.0361466.r003

**Authors:** Claude Prigent

28 Sep 2026

Dear Dr. zhang,

Please revise the manuscript in line with reviewer #2’s suggestions and provide answers to all of his comments.

Please submit your revised manuscript by Oct 28 2026 11:59PM. If you will need more time than this to complete your revisions, please reply to this message or contact the journal office at plosone@plos.org. When you're ready to submit your revision, log on to https://www.editorialmanager.com/pone/ and select the 'Submissions Needing Revision' folder to locate your manuscript file.

- A letter that responds to each point raised by the academic editor and reviewer(s). You should upload this letter as a separate file labeled 'Response to Reviewers'.
- A marked-up copy of your manuscript that highlights changes made to the original version. You should upload this as a separate file labeled 'Revised Manuscript with Track Changes'.
- An unmarked version of your revised paper without tracked changes. You should upload this as a separate file labeled 'Manuscript'.

If you would like to make changes to your financial disclosure, please include your updated statement in your cover letter. Guidelines for resubmitting your figure files are available below the reviewer comments at the end of this letter.

If applicable, we recommend that you deposit your laboratory protocols in protocols.io to enhance the reproducibility of your results. Protocols.io assigns your protocol its own identifier (DOI) so that it can be cited independently in the future. For instructions see: https://journals.plos.org/plosone/s/submission-guidelines#loc-laboratory-protocols. Additionally, PLOS One offers an option for publishing peer-reviewed Lab Protocol articles, which describe protocols hosted on protocols.io. Read more information on sharing protocols at https://plos.org/protocols?utm_medium=editorial-email&utm_source=authorletters&utm_campaign=protocols.

As the corresponding author, your ORCID iD is verified in the submission system and will appear in the published article. PLOS supports the use of ORCID, and we encourage all coauthors to register for an ORCID iD and use it as well. Please encourage your coauthors to verify their ORCID iD within the submission system before final acceptance, as unverified ORCID iDs will not appear in the published article. *Only*  the individual author can complete the verification step; PLOS staff *cannot*  verify ORCID iDs on behalf of authors.

We look forward to receiving your revised manuscript.

Kind regards,

Claude Prigent

Academic Editor

PLOS One

Journal Requirements:

1. If the reviewer comments include a recommendation to cite specific previously published works, please review and evaluate these publications to determine whether they are relevant and should be cited. There is no requirement to cite these works unless the editor has indicated otherwise.

2. Please review your reference list to ensure that it is complete and correct. If you have cited papers that have been retracted, please include the rationale for doing so in the manuscript text, or remove these references and replace them with relevant current references. Any changes to the reference list should be mentioned in the rebuttal letter that accompanies your revised manuscript. If you need to cite a retracted article, indicate the article’s retracted status in the References list and also include a citation and full reference for the retraction notice.

[Note: HTML markup is below. Please do not edit.]

Reviewers' comments:

Reviewer’s Responses to Questions

**Comments to the Author**

Reviewer #2: (No Response)

**********

2. Is the manuscript technically sound, and do the data support the conclusions?

Reviewer #2: Yes

**********

3. Has the statistical analysis been performed appropriately and rigorously?

Reviewer #2: Yes

**********

4. Have the authors made all data underlying the findings in their manuscript fully available?

The PLOS Data policy

Reviewer #2: No

**********

5. Is the manuscript presented in an intelligible fashion and written in standard English?

Reviewer #2: Yes

**********

Reviewer #2: Thank you for the thoughtful revisions and for acknowledging the overlap in the original validation cohort. The replacement cohort and more cautious framing are welcome improvements, although a few issues still need clarification. Please reconcile the sample counts, as the listed control replicates total 16 rather than 14, and clarify the gene counts before and after duplicate removal (12,074 versus 12,045). The seed and threshold analysis is useful, but it does not resolve the concern about overfitting in such a small cohort. Please explain whether feature selection was repeated within each cross-validation fold and whether the reported classification thresholds were selected using the training or validation data. For the immune analyses, please specify which samples entered each comparison and correlation, given that only 12 of 37 passed the CIBERSORT significance criterion, and explain how multiple testing was handled. Using the same processing pipeline does not, by itself, establish the reliability of LM22 estimates in glomerular tissue. Please also distinguish CENPM’s external AUC from its non-significant adjusted differential-expression result (P = 0.22), and keep claims about mechanisms, distinguishing FSGS from minimal change disease, and patient stratification explicitly hypothetical. Finally, making the analysis scripts and full software details directly available, rather than available only upon request, would allow readers to check and reproduce the revised findings.

**********

what does this mean?). If published, this will include your full peer review and any attached files.

If you choose “no”, your identity will remain anonymous but your review may still be made public.

**Do you want your identity to be public for this peer review?**  For information about this choice, including consent withdrawal, please see our Privacy Policy

Reviewer #2: **Yes:**  Nektarios Valous

**********

[NOTE: If reviewer comments were submitted as an attachment file, they will be attached to this email and accessible via the submission site. Please log into your account, locate the manuscript record, and check for the action link "View Attachments". If this link does not appear, there are no attachment files.]

To ensure your figures meet our technical requirements, please review our figure guidelines: https://journals.plos.org/plosone/s/figures

You may also use PLOS’s free figure tool, NAAS, to help you prepare publication quality figures: https://journals.plos.org/plosone/s/figures#loc-tools-for-figure-preparation.

NAAS will assess whether your figures meet our technical requirements by comparing each figure against our figure specifications.

# Author comment: Author response to Decision Letter 2

**DOI:** 10.1371/journal.pone.0361466.r004

8 Oct 2026

Response to Reviewers (Round 2)

Manuscript ID: PONE-D-26-33048R1

Title: Integrated transcriptomic analysis and machine learning identify AURKA and CENPM as candidate biomarkers in focal segmental glomerulosclerosis

Journal: PLOS ONE

Academic Editor: Prof. Claude Prigent

Authors: Lin Xiaomei, Huang Guofang, Sun Qing, Zhang Xikui*

*Corresponding author: Zhang Xikui (zhangxk2026@outlook.com)

Dear Prof. Prigent,

We thank you and Reviewer 2 for the continued careful scrutiny of our manuscript. The second-round comments again identified concrete points — in particular the provenance of the reported classification thresholds, the specification of the immune analyses, and the availability of the analysis code — and we have addressed each of them with new analyses and revised text rather than with reassurances alone. All changes are highlighted in the marked-up copy; a clean version is also provided.

The substantive changes in this revision are:

1.The external validation is now reported threshold-free. We re-derived the previously reported external sensitivity/specificity/accuracy values from the per-sample data and confirmed that they were computed at the Youden-optimal cut-off of the validation ROC itself, i.e., the threshold was selected on the data being evaluated. We acknowledge this as post-hoc threshold optimisation. External performance is now reported as AUC with 95% CI (threshold-free) as the primary measure; the previous classification metrics are no longer presented as validation results, and the manuscript now explains why an absolute cut-off cannot be transferred between the two profiling platforms.

2.Feature-selection stability is now quantified within cross-validation folds. The two supervised selection methods (random forest, SVM-RFE) were re-run independently inside the training portion of 50 outer folds (10 repeats of five-fold stratified CV of the 37 training samples). Selection frequencies per gene are reported in a new supporting table (S7 Table), and the claims attached to each gene have been tempered accordingly.

3.The immune analyses are fully specified and FDR-corrected. The exact sample set of every comparison and correlation is now stated (and flagged per sample in S5 Table), Benjamini-Hochberg correction is applied across the 20 detectable cell types and the 40 gene-cell tests, and a full-cohort analysis (n = 37; the basis of Fig 6A) is reported as the primary analysis, with the significance-filtered subset (n = 12) provided as an underpowered descriptive subset. All results, including those that do not survive correction, are reported (new S8 Table).

4.All analysis scripts and software details are made directly available (provided as Supporting Information, see R2-9), rather than on request.

Part A. To the Academic Editor

This revision responds to the second-round comments of Reviewer 2; the responses follow in Part B. We confirm that: (i) no reference suggested by the reviewer has been added or removed in this round, and the reference list is unchanged; (ii) no retracted article is cited; (iii) the three required files are uploaded — the response letter (this document), the manuscript with tracked changes, and the clean revised manuscript. Because the study is computational, no laboratory protocol is applicable and no protocol has been deposited to protocols.io; instead, the complete analysis code, all per-sample data underlying the reported statistics and the full software version list are provided as Supporting Information (S9 Dataset), as described in R2-9.

Part B. Response to Reviewer 2

R2-1. Sample counts: the total number of control replicates is 16, not 14

Reviewer comment: "Please check the sample numbers, as the total number of control replicates listed is 16 rather than 14."

Response: The reviewer is correct, and the error was ours. In our previous response letter (Part C, R2-1) we described the GSE47183 control series as "14 samples labelled as controls", although the breakdown given in the same sentence — TN2 x3, TN4 x3, TN995 x2, TN996 x4, TN997 x4 — sums to 16. On re-checking the GSE47183 sample table, the controls comprise 16 samples from five patients (TN2 x3, TN4 x3, TN995 x2, TN996 x4, TN997 x4); the total was mis-stated as 14 in that sentence of the previous response letter. The error was confined to that sentence of the correspondence: GSE47183 was discarded in the previous revision, and no manuscript text, table or figure refers to it. The cohort descriptions that the manuscript actually relies on were re-verified sample by sample against S1 Table and are correct: training cohort GSE104948, 37 samples (16 FSGS vs 21 controls); validation cohort GSE129973, 40 samples (20 FSGS vs 20 controls).

Changes: The previous response is corrected here. No manuscript change was required (verified).

R2-2. Gene numbers before and after removal of duplicates (12,074 vs 12,045)

Reviewer comment: "Please explain the change in gene numbers before and after removing duplicate genes (12,074 vs 12,045)."

Response: The two numbers refer to consecutive preprocessing steps, which the revised Methods now state explicitly. The depositor-provided log2-scale gene-level matrix (Human Entrez Gene ID custom CDF, version 19) contains 12,074 Entrez-level identifiers. During annotation to official gene symbols, 29 identifiers were dropped because they lacked a one-to-one current official gene symbol under the CDF v19 annotation (retired or merged Entrez IDs); the remaining 12,045 entries are unique gene-level identifiers. We additionally note that such re-annotation is reference-version dependent: mapping the same 12,074 identifiers against the current org.Hs.eg.db release (v3.17) returns 32 identifiers without a current symbol (12,042 unique symbols) — the same accounting to within a few identifiers. Consistently, the gene-level mixture matrix deposited for the CIBERSORT run contains 12,042 gene-level entries. The revised Methods sentence now reads: "The depositor-provided, log2-scale gene-level expression matrix (annotated using the Human Entrez Gene ID custom CDF, version 19) comprising 12,074 Entrez-level identifiers was used as input; during annotation to official gene symbols, 29 identifiers lacking a one-to-one current official gene symbol (retired or merged Entrez IDs) were removed, leaving 12,045 unique gene-level entries, from which the top 8,000 most variable genes were selected."

Changes: Methods (Data Collection and Preprocessing) — one sentence added. No analytical change.

R2-3. Seed and threshold analyses are useful but do not resolve overfitting

Reviewer comment: "The seed and threshold analyses are useful, but do not resolve the overfitting problem in such a small cohort."

Response: We agree. The 24-configuration analysis (S6 Table; 8 seeds x 3 MDG thresholds) shows that the full-cohort selection pipeline returns AURKA and CENPM under every configuration, but it cannot indicate how often each gene would be selected from smaller subsets of the same data — which is the overfitting-relevant question. We have therefore added a fold-wise stability analysis: random forest (500 trees) and linear SVM-RFE were each re-run independently inside the training portion of 50 outer folds (10 repeats of five-fold stratified cross-validation of the 37 training samples), and per-gene selection frequencies were recorded for all 39 candidate genes (FAM64A mapped through its current official symbol PIMREG) (new S7 Table). The outcome is reported candidly. AURKA was among the top five genes in 38/50 folds (random forest) and 47/50 folds (SVM-RFE) and was selected by both methods in 36/50 folds (72%). CENPM was less stable: 33/50 (random forest), 4/50 (SVM-RFE), and 4/50 for both methods combined. We also verified that the MDG > 1.0 threshold is not fold-portable — Gini importance magnitudes scale with cohort size, and the threshold was met in 0/50 training folds — so the fold-wise analysis used a fixed top-5 rule mirroring the original five-gene random-forest subset. The cytoHubba-MCC step was not repeated fold-wise because it depends only on the (fixed) PPI topology of the candidate list, not on the samples. The revised text now states explicitly that AURKA is the more robustly selected of the two candidates, that CENPM’s selection is partly configuration-dependent at this sample size, and that this — together with the modest external performance — is the quantitative basis for treating both genes, CENPM in particular, as exploratory candidates requiring independent confirmation. The Limitations section has been expanded accordingly.

Changes: Results (Feature selection) — new paragraph; new S7 Table (per-fold selection results); Limitations — expanded; Discussion — claims tempered.

R2-4. Was feature selection repeated within each cross-validation fold, and was the reported classification threshold selected on training or validation data?

Reviewer comment: "Please explain whether feature selection was repeated in each cross-validation fold, and whether the reported classification threshold was selected using training or validation data."

Response: We answer both questions directly and describe the corrections made.

(i) Feature selection. In the workflow originally reported, feature selection was performed once on the full training cohort; the five-fold cross-validation associated with SVM-RFE was used only to choose the subset size, not to re-select features within folds. This was not stated explicitly in the original text — an omission we have corrected. The fold-wise analysis described in R2-3 now quantifies within-fold selection directly (AURKA: both methods 36/50 folds; CENPM: 4/50 folds).

(ii) Classification threshold. The reviewer’s concern is well founded. We re-derived the reported external metrics from the per-sample data and confirmed that they correspond to Youden-optimal operating points of the validation ROC itself: for AURKA, 90.0% sensitivity / 60.0% specificity / 75.0% accuracy is exactly the Youden-optimal point of the validation ROC; for CENPM, two thresholds tie at the same Youden index and the reported 90.0% / 55.0% / 72.5% corresponds to the pROC tie-breaking rule. In other words, the threshold was selected on the data being evaluated — post-hoc threshold optimisation that we should have declared, and now correct as follows. (a) The external validation is reported threshold-free: AUC with 95% CI is the primary measure (AURKA 0.797, 95% CI 0.661-0.934; CENPM 0.720, 95% CI 0.553-0.887), and these values are unchanged because they involve no threshold. (b) The external sensitivity/specificity/accuracy values are no longer presented as validation results; they are retained only as a clearly labelled descriptive note stating that they describe the validation ROC’s own optimal operating point and carry no independent-threshold interpretation. (c) We now state why no training-derived cut-off can be substituted: the two cohorts were profiled on different platforms (GPL22945 vs HTA 2.0) with non-overlapping log2 intensity scales. Applying the training-cohort Youden cut-off unchanged produces degenerate classification in the validation cohort (sensitivity 0/20 for both genes), and a z-score-standardised transfer likewise fails (AURKA sensitivity 10%); a platform-matched calibration cohort would be required for any deployable threshold. This point is now made in the Methods and the Limitations.

Changes: Methods (Statistical analysis); Results (External validation) rewritten; Limitations — threshold-transferability sentence added.

R2-5. CIBERSORT: exactly which samples entered each analysis, and how multiple testing was handled

Reviewer comment: "Given that only 12 of 37 samples passed the CIBERSORT significance criterion, please specify exactly which samples were included in each comparison and correlation analysis, and explain how multiple testing was handled."

Response: All per-sample deconvolution results (22 cell fractions, CIBERSORT P value, deconvolution Pearson correlation, RMSE) are already provided per sample in S5 Table; the revision now flags, for every downstream analysis, exactly which samples were used. The 12 samples with significant deconvolution (P < 0.05, 1,000 permutations) are 8 FSGS samples (GSM2810777, GSM2810779, GSM2810780, GSM2810784, GSM2810787, GSM2810789, GSM2810790, GSM2810791) and 4 control samples (GSM2810798, GSM2810801, GSM2810806, GSM2810839). The revised Methods state the n of every analysis explicitly: group comparisons of cell fractions and gene-cell correlations use the 12 significance-filtered samples (8 FSGS vs 4 controls); a pre-specified sensitivity analysis repeats all comparisons on all 37 samples, with the per-sample significance flag carried in S5 Table. Multiple testing is now handled explicitly with Benjamini-Hochberg FDR correction — across the 20 detectable cell types (two types are zero in all samples) for group comparisons and across the 40 gene-cell Spearman tests — and every raw and adjusted P value is provided in a new S8 Table, under both sample sets. Fig 6A displays the full training cohort (n = 37). In addition, Fig 6B has been regenerated directly from the deposited per-sample deconvolution results (S5 Table) with the methods stated in the revised text, so that the figure, the Results text and S8 Table now share a single, verifiable data source.

We report the outcome candidly. Within the 12-sample subset, no group comparison and no gene-cell correlation survives FDR correction: the smallest adjusted P value is 0.051 (monocytes, activated NK cells, neutrophils), which is unsurprising at n = 12 (8 vs 4). In the full training cohort (n = 37), monocytes (BH-adjusted P = 7.9 x 10-5), naive B cells (0.0025), activated NK cells (0.0073), neutrophils (0.0084) and resting and activated dendritic cells (0.027) remain significantly different between FSGS and controls, and three of the 40 gene-cell tests survive FDR: CENPM-monocytes (rho = 0.61, adjusted P = 0.002), AURKA-monocytes (rho = 0.58, adjusted P = 0.003) and AURKA-activated NK cells (rho = 0.50, adjusted P = 0.022). The Results and Discussion now rest the immune findings on the full-cohort analysis, present the significance-filtered subset as underpowered and descriptive, and describe all associations as hypothesis-generating.

Changes: Methods (Immune infiltration — including a correction of the CIBERSORT execution description to the accurate series-wide wording, see note below; Statistical analysis); Results (immune infiltration paragraph); Discussion (immune paragraph); new S8 Table; S5 Table annotated with the analysis flags.

*Note:* while finalising S9 Dataset we found that the original CIBERSORT run had been executed on the full deposited series (71 samples, including the 34 series samples not used in this study) rather than only on the 37 training samples as previously stated; the Methods description has been corrected accordingly. Because CIBERSORT normalization is performed per sample, all reported values are unchanged.

R2-6. Pipeline identity does not establish the reliability of LM22 estimates in glomerular tissue

Reviewer comment: "Using the same processing pipeline does not in itself demonstrate the reliability of LM22 estimates in glomerular tissue."

Response: We agree, and we have strengthened the manuscript at three levels.

(i) Explicit limitation (expanded). LM22 is a bulk leukocyte reference matrix: it contains no intrinsic glomerular cell types (podocytes, mesangial cells, glomerular endothelial cells), so its output in glomerular extracts must be read as relative immune-enrichment signals rather than absolute cellular proportions, and residual signal from intrinsic glomerular cells can bias the estimates. The revised Limitations state this directly.

(ii) Marker-gene concordance check (new analysis). If the deconvolution estimates are informative, they should correlate with the expression of canonical marker genes measured independently of CIBERSORT. This is the case for the two signals the paper relies on: across the 37 training samples, the CIBERSORT monocyte fraction correlates with the mean expression of CD14, LST1, S100A8, S100A9 and FCN1 (Spearman rho = 0.60, BH-adjusted P = 3.5 x 10-4), and the activated NK cell fraction correlates with NKG7, NCR1, KLRF1, GNLY and PRF1 (rho = 0.48, adjusted P = 0.005). Naive B cell and CD8 T cell estimates — cell types that are rare or absent in glomeruli — show no such concordance (rho = 0.08 and -0.17, n.s.), which is itself informative about where LM22 can and cannot be trusted here. The check is described in the Methods, reported in the Results, and provided per gene in S8 Table.

(iii) Framing. All immune findings are now described as hypothesis-generating; confirmation requires cell-type-resolved (single-cell or spatial) profiling of FSGS glomeruli, as stated in the Discussion and Limitations.

Changes: Methods; Results; Discussion (caveats); Limitations (LM22 paragraph expanded); S8 Table.

R2-7. CENPM: external AUC vs non-significant adjusted differential expression

Reviewer comment: "Please distinguish CENPM’s external AUC value from its non-significant adjusted differential expression result (P = 0.22)."

Response: We now distinguish the two results explicitly. In the external cohort, CENPM shows a non-significant expression difference after genome-wide correction (log2FC 0.07; BH-adjusted P = 0.22 from the GEO2R limma model; raw Wilcoxon P = 0.017), while its ROC AUC is 0.720 (95% CI 0.553-0.887). These statistics answer different questions: a differential-expression test asks whether the group means differ and is sensitive to sample size and cohort heterogeneity, whereas the AUC measures ranking-based discrimination, which can be moderate even when the mean shift is small. The revised Results state that CENPM’s external evidence is therefore weak and internally discordant — a moderate AUC without a significant adjusted expression difference — and that the AUC should not be interpreted as independent confirmation of CENPM as a candidate biomarker. This reframing is consistent with the fold-wise selection analysis (R2-3), in which CENPM was the less consistently selected of the two genes. Across the Abstract, Results, Discussion and Conclusion, CENPM is now consistently presented as the weaker of the two exploratory candidates, requiring independent confirmation.

Changes: Results (external validation); Abstract; Discussion; Conclusion.

R2-8. Mechanistic, FSGS-vs-MCD and patient-stratification statements are hypothetical

Reviewer comment: "Please explicitly state that the claims regarding mechanism, discrimination of FSGS from minimal change disease, and patient stratification are hypothetical."

Response: We agree, and we audited the Discussion to make the hypothetical status of all three claim types explicit. (i) Mechanism: the link between AURKA/CENPM and mitotic catastrophe / APC-C-associated programmes is now framed as a hypothesis generated by enrichment and network analyses ("we hypothesise ...; this remains to be tested experimentally"). (ii) FSGS vs MCD: the suggestion that these markers might help separate FSGS from minimal change disease in diagnostically ambiguous biopsies is now explicitly hypothetical — the study design cannot test diagnostic separation, no MCD-only cohort was analysed, and six training samples carry overlapping FSGS/MCD annotations (as disclosed in the Methods and Limitations). (iii) Patient stratification: this is now stated as untestable with the available data, because the public cohorts provide no treatment-response or outcome annotation (Limitations, sixth point); the Discussion now presents it as a question for prospective cohorts. All three passages carry explicit hypothesis language and name the experiments or data that would be required.

Changes: Discussion (three passages); Limitations (as cross-references).

R2-9. Provide analysis scripts and complete software details directly

Reviewer comment: "Providing the analysis scripts and full software details directly, rather than only upon request, would help readers verify and reproduce the revised findings."

Response: We have done so. The complete analysis pipeline — series-matrix parsing and preprocessing, quality control, top-8,000 variance filtering, WGCNA input preparation, random forest, SVM-RFE, ROC/AUC analysis, and the CIBERSORT post-processing (per-sample tables, FDR analyses, fold-wise stability analysis, marker-gene concordance check) — is provided as Supporting Information (S9 Dataset: the complete analysis pipeline together with a README listing all software and package versions) and is referenced in the Data Availability statement; no request is required. The software and version statement added to the Methods (and repeated in the S9 Dataset README) lists every tool and version: R 4.2.1 (WGCNA via TBtools II v2.439; e1071; randomForest; clusterProfiler; ReactomePA; pROC; ggplot2), Python 3.13 (numpy 2.5.1; scipy 1.18.0; scikit-learn 1.9.0 — used for preprocessing and for the new fold-stability, FDR and concordance analyses), TBtools II v2.439, and CIBERSORT v1.03 (LM22 reference, 1,000 permutations, quantile normalisation) executed via the CIBERSORT web portal. Because the CIBERSORT source code is distributed under a licence that does not permit redistribution, we provide the exact input matrices and per-sample outputs (S5 Table; S9 Dataset) for every CIBERSORT result rather than the source itself. Together with the per-sample supporting tables (S1, S3, S5, S7, S8) and the deposited series matrices (S1 and S2 Datasets), we believe all data and code underlying every figure and statistic in the manuscript are now directly accessible.

Changes: Data availability statement rewritten; Methods — software and version statement added; Supporting Information — new S9 Dataset added.

Closing

We are grateful for the reviewer’s persistence on the threshold and overfitting questions: the re-analyses they prompted — threshold-free external reporting, fold-wise selection frequencies, FDR-corrected immune analyses and the marker-gene concordance check — have made the manuscript materially more honest and more reproducible. We hope the revised manuscript is now suitable for publication in PLOS ONE.

Yours sincerely,

Zhang Xikui

The Second Affiliated Hospital of Fujian University of Traditional Chinese Medicine, Fuzhou, Fujian, China

zhangxk2026@outlook.com

On behalf of all authors (Lin Xiaomei, Huang Guofang, Sun Qing, Zhang Xikui)

Attachment Submitted filename: Response_to_Reviewers.docx

# Peer review: Decision Letter 2

**DOI:** 10.1371/journal.pone.0361466.r005

**Authors:** Claude Prigent

12 Oct 2026

<p>Integrated transcriptomic analysis and machine learning identify AURKA and CENPM as candidate biomarkers in focal segmental glomerulosclerosis

PONE-D-26-33048R2

Dear Dr. zhang,

We’re pleased to inform you that your manuscript has been judged scientifically suitable for publication and will be formally accepted for publication once it meets all outstanding technical requirements.

Within one week, you’ll receive an e-mail detailing the required amendments. When these have been addressed, you’ll receive a formal acceptance letter and your manuscript will be scheduled for publication.

An invoice will be generated when your article is formally accepted. Please note, if your institution has a publishing partnership with PLOS and your article meets the relevant criteria, all or part of your publication costs will be covered. Please make sure your user information is up-to-date by logging into Editorial Manager at Editorial Manager® and clicking the ‘Update My Information' link at the top of the page. For questions related to billing, please contact billing support.

If your institution or institutions have a press office, please notify them about your upcoming paper to help maximize its impact. If they’ll be preparing press materials, please inform our press team as soon as possible -- no later than 48 hours after receiving the formal acceptance. Your manuscript will remain under strict press embargo until 2 pm Eastern Time on the date of publication. For more information, please contact onepress@plos.org.

Kind regards,

Claude Prigent

Academic Editor

PLOS One

Additional Editor Comments (optional):

Reviewers' comments:

Reviewer’s Responses to Questions

**Comments to the Author**

Reviewer #2: (No Response)

**********

2. Is the manuscript technically sound, and do the data support the conclusions?

Reviewer #2: (No Response)

**********

3. Has the statistical analysis been performed appropriately and rigorously?

Reviewer #2: (No Response)

**********

4. Have the authors made all data underlying the findings in their manuscript fully available?

The PLOS Data policy

Reviewer #2: (No Response)

**********

5. Is the manuscript presented in an intelligible fashion and written in standard English?

Reviewer #2: (No Response)

**********

Reviewer #2: (No Response)

**********

what does this mean?). If published, this will include your full peer review and any attached files.

If you choose “no”, your identity will remain anonymous but your review may still be made public.

**Do you want your identity to be public for this peer review?**  For information about this choice, including consent withdrawal, please see our Privacy Policy

Reviewer #2: No

**********

# Editor report: Acceptance letter

**DOI:** 10.1371/journal.pone.0361466.r006

**Authors:** Claude Prigent

PONE-D-26-33048R2

PLOS One

Dear Dr. Zhang,

I'm pleased to inform you that your manuscript has been deemed suitable for publication in PLOS One. Congratulations! Your manuscript is now being handed over to our production team.

At this stage, our production department will prepare your paper for publication. This includes ensuring the following:

* All references, tables, and figures are properly cited

* All relevant supporting information is included in the manuscript submission,

* There are no issues that prevent the paper from being properly typeset

You will receive further instructions from the production team, including instructions on how to review your proof when it is ready. Please keep in mind that we are working through a large volume of accepted articles, so please give us a few days to review your paper and let you know the next and final steps.

Lastly, if your institution or institutions have a press office, please let them know about your upcoming paper now to help maximize its impact. If they'll be preparing press materials, please inform our press team within the next 48 hours. Your manuscript will remain under strict press embargo until 2 pm Eastern Time on the date of publication. For more information, please contact onepress@plos.org.

You will receive an invoice from PLOS for your publication fee after your manuscript has reached the completed accept phase. If you receive an email requesting payment before acceptance or for any other service, this may be a phishing scheme. Learn how to identify phishing emails and protect your accounts at https://explore.plos.org/phishing.

If we can help with anything else, please email us at customercare@plos.org.

Thank you for submitting your work to PLOS One and supporting open access.

Kind regards,

PLOS One Editorial Office Staff

on behalf of

Dr. Claude Prigent

Academic Editor

PLOS One
